# A Decade of Progress: Review of the Synthesis of Dibenzo[*b*,*f*]oxepines

**DOI:** 10.1002/tcr.202500050

**Published:** 2025-10-06

**Authors:** Gabriel Pereira da Costa, Bruna Rafaella Schneider, Angelita Manke Barcellos, Liane Krolow Soares, Rafael Centurião Brinkerhoff, Fernanda Piecha Ludwig, Alex Fabiani Claro Flores

**Affiliations:** ^1^ Núcleo de Estudo Estrutural e Síntese de Heterociclos ‐ NEESH Universidade Federal do Rio Grande ‐ FURG Escola de Química e Alimentos ‐ EQA, Itália Ave., km 8, Campus Carreiros Rio Grande 96.203‐900 RS Brazil; ^2^ Pesquisa em Síntese Orgânica Sustentável ‐ PSOS Universidade Federal do Rio Grande ‐ FURG Escola de Química e Alimentos ‐ EQA, Itália Ave., km 8, Campus Carreiros Rio Grande 96.203‐900 RS Brazil

**Keywords:** dibenzo[*b*,*f*]oxepines, O‐heterocycles, oxepine ring construction, pharmaceutical applications, seven‐membered heterocycles

## Abstract

This review article highlights the significant advances in the synthesis of dibenzo[*b*,*f*]oxepines over the past decade. Dibenzo[*b*,*f*]oxepines, important heterocyclic compounds, have attracted increasing interest due to their wide‐ranging applications in medicinal chemistry and materials applications. The review addresses traditional approaches and recent developments, highlighting efficient synthetic strategies such as cross‐coupling reactions, intramolecular cyclizations, and molecular diversification strategies. Additionally, the efficiency, selectivity, and sustainability of these methods are discussed. Emerging trends and future challenges in the synthesis of dibenzo[*b*,*f*]oxepines are also explored, including the search for more sustainable methods, the expansion of structural diversity, and the optimizing reaction conditions. This review provides a comprehensive overview of recent advances in this field, providing valuable insights for researchers aiming to develop novel synthetic strategies and applications for dibenzo[*b*,*f*]oxepines.

## Graphical abstract

This review summarizes recent advances in dibenzo[*b*,*f]*oxepine synthesis, highlighting medicinal and materials applications. It discusses classical and modern methods, including cross‐coupling, intramolecular cyclizations, and diversification strategies, emphasizing efficiency, selectivity, and sustainability. Emerging trends such as greener approaches, structural diversity, and optimized conditions are addressed, providing researchers with valuable insights into innovative synthetic strategies and potential applications.

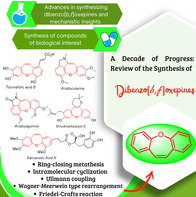

## 1 Introduction

The importance of heterocyclic compounds is unquestionable, as they are structural units found in most natural and synthetic bioactive compounds, pharmaceuticals, and agrochemicals. They can also be used as synthetic organic intermediates to obtain compounds with more complex structures.^[^ ^1^ ^]^ These compounds are characterized by the presence of one or more (hetero)atoms, which may be the same or different from each other, within a cyclic carbon framework. The most common heteroatoms are nitrogen, oxygen, and sulfur.^[^ ^2^ ^]^

**Figure 1:**
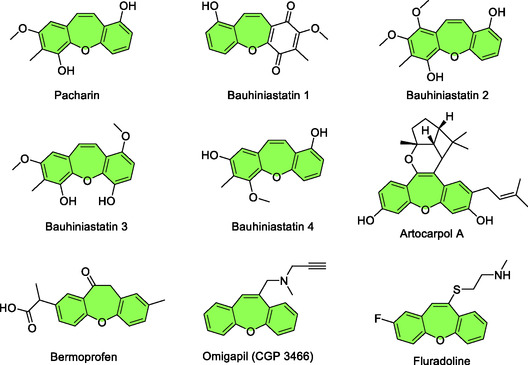
Examples of natural products containing dibenzo[*b*,*f*]oxepine unit.

In this sense, a 7‐membered heterocycle containing an oxygen atom, such as oxepines ‐ especially dibenzo[*b*,*f*]oxepines ‐ stand out, as they are structures found in both natural products and synthetic compounds of significant interest (**Figure** **1**). This structural unit is present in natural compounds that exhibit a wide range of pharmacological activities, such as Pacharin and Bauhiniastatin **1–4**, which show inhibitory activity on the growth of cancer cells. It is also found in the structure of CGP 3466, used in the treatment of Parkinson's disease, in Artocarpol A and Bermoprofen, which have anti‐inflammatory activity, and in Fluradoline, known for its analgesic effects, among others (Figure 1).^[^ ^3^ ^]^

**Scheme 1:**
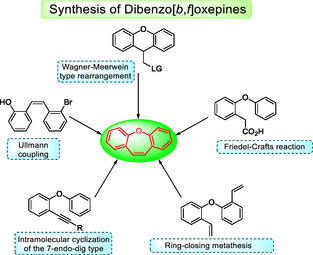
Several strategies to synthesize the dibenzo[*b*,*f*]oxepine unit.

There are several well‐established methods for the synthesis of oxepines.^[^ ^4^ ^]^ One possible synthetic pathway involves intramolecular Friedel‐Crafts reactions using functionalized diaryl ethers. A approach uses xanthene substituted with leaving groups which undergo a Wagner‐Meerwein‐type rearrangement.^[^ ^5^ ^]^ Over the years, additional strategies have been developed, such as Ullmann coupling of stilbenes, ring‐closing metathesis (RCM)^[^ ^6^ ^]^ using 2,2′‐oxybis(vinylaryl) compounds, and more recently, 7‐endo‐dig intramolecular cyclization reactions of *o*‐phenoxy diarylacetylenes promoted by Lewis acid (**Scheme** **1**).^[^ ^7^ ^]^

In addition to the aforementioned aspects, these compounds show high potential for application in photoactive materials, given the increasing number of patent filings involving this scaffold with such properties. Dibenzo[b,f]oxepines are particularly notable for their ability to switch from a “V''‐shaped conformation to a planar conformation in the excited state, as well as having a large Stokes shift and a well‐defined vibrational structure in the fluorescence spectrum.^[^ ^8^ ^]^

Prior to 2013, a variety of synthetic strategies were developed, including Pd‐catalyst processes,^[^ ^9^ ^]^ ring expansion reaction,^[^ ^10^ ^]^ oxygen performing nucleophilic aromatic substitution.^[^ ^11^ ^]^ along with methods for the synthesis of cyclic systems from natural products such as *Artocarpol A* and *D* and compounds with biological interest has also been reported in the literature.^[^ ^12^ ^]^ More recently, some methods use the previously synthesized dibenzo[*b*,*f*]oxepine unit containing a reactive group to carry out successive reactions, as described by Krawczyk and coworkers in 2018 and 2022.^[^ ^13^ ^]^ In 2022, Brahmachari and coworkers^[^ ^14^ ^]^ published a comprehensive review of the biologically promising natural oxepine compounds, highlighting their potential in diverse therapeutic areas. The following year (2023), Krawczyk published another review addressing various structural features and pharmacological actions of dibenzo[*b*,*f*] oxepine derivatives,^[^ ^15^ ^]^ while Marais and coworkers reported various synthetic strategies for accessing dibenzo[*b*,*f*]heteropines.^[^ ^16^ ^]^ However, despite these contributions,^[^ ^17^ ^]^ there remains a gap in the literature regarding systematic approaches that consolidate different synthetic methodologies and elucidate reaction mechanisms. In this regard, the focus of this review is to provide a comprehensive overview of synthetic strategies developed since 2013 for the construction of the dibenzo[*b*,*f*]oxepine scaffold, including detailed descriptions of the methods and proposed mechanisms.

## 2 Protocols for Synthesizing Dibenzo[*b*,*f*]oxepine

In this section, several protocols for obtaining the target dibenzo[*b*,*f*]oxepine are presented, organized in chronological order of publication. The reaction mechanisms and peculiarities of each study are discussed in detail. This section provides a comprehensive overview of recent developments in the synthesis of dibenzo[*b*,*f*]oxepines.

### 2.1 Protocols for Synthesizing Dibenzo[*b*,*f*]oxepine from 2013 to 2016

**Scheme 2:**
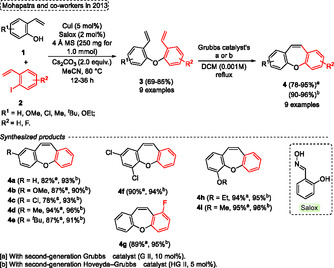
Synthesis of dibenzo[*b*,*f*]oxepine unit described by Mohapatra and coworkers^[6b]^ in 2013.

Mohapatra and coworkers^[6b]^ described in 2013, advances in the synthesis of dibenzo[*b*,*f*]oxepine scaffolds **4**, which were obtained by a two‐step approach. The first step involves an Ullmann‐type reaction to afford the etheration compound, and the tricyclic **4** target compounds are obtained by subsequent RCM reactions. In this protocol, initially, different 2‐vinyl phenols **1** were reacted with iodo‐2‐vinylbenzenes **2** in the presence of CuI (5 mol%) as the catalyst, Salox (2 mol%) as ligand, 4 Å MS (250 mg for 1.0 mmol), 2 equiv. of Cs_2_CO_3_ as base, MeCN. The reactions were carried out at 80 °C for 12–36 h, yielding diaryl‐ether products **3** (9 examples) in good yields (69%–85%) (**Scheme** **2**).

The intermediate compounds **3** were applied in RCM reactions using a second‐generation Grubbs catalyst (G II, 10 mol%), or second‐generation Hoveyda–Grubbs catalyst (HG II, 5 mol%) in the presence of DCM as solvent at reflux temperature. As reported by the authors, the reaction was conducted under high‐dilution conditions (0.001 M) to avoid a dimerization reaction. The conditions described above afforded the target dibenzo[*b*,*f*]oxepine **4** (9 examples) excellent yields for both catalysts (G II or HG II) with good tolerance of various functional groups. In general, the second‐generation Hoveyda–Grubbs catalyst (HG II) gave better yields of desired compounds **4** (90%–96%) when compared to the second‐generation Grubbs catalyst (G II) (78%–95%) (Scheme 2).

**Scheme 3:**
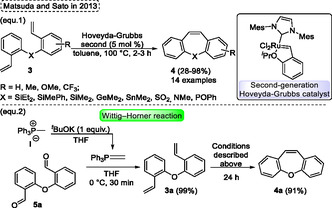
Synthesis of dibenzo[*b*,*f*]oxepine unit described by Matsuda and Sato^[6a]^ in 2013.

Similarly, in 2013, Matsuda and Sato^[6a]^ reported the synthesis of various dibenzo[*b*,*f*]heteropines **4** containing substituents from group 13–16 elements (such as SiEt_2_, SiMePh, SiMe_2_, GeMe_2_, SnMe_2_, SO_2_, NMe, and POPh) via RCM in the presence of second‐generation Hoveyda−Grubbs catalyst (**Scheme** **3**, eq. 1). Their methodology was also efficiently extended to obtention of dibenzo[*b*,*f*]oxepine **4a**. In this regard, to obtain the intermediate methylenetriphenylphosphorane to perform the Wittig–Horner reaction, the MePPh_3_I underwent a reaction with 1 equiv. of ^*t*^BuOK, THF as solvent. Subsequently, bis(2‐formylphenyl) ether **5a** was added to this mixture and stirred at 0 °C for 30 min, leading to the formation of compound **3a** with a high yield (99%). The resulting bis(2‐vinylphenyl) ether **3a** was then subjected to the presence of 5 mol% of Hoveyda–Grubbs second‐generation in toluene at 100 °C for 24 h affording the desired RCM product **4a** in 91% yield (Scheme 3, eq. 2).

**Scheme 4:**
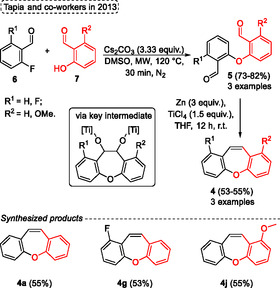
Synthesis of dibenzo[*b*,*f*]oxepine unit described by Tapia and coworkers^[^ ^18^ ^]^ in 2013.

Several protocols involve a nucleophilic aromatic substitution (S_N_Ar) followed by a condensation step to obtain the desired dibenz[*b*,*f*]oxepine derivatives **4**. In 2013, Tapia and coworkers^[^ ^18^ ^]^ reported the intermolecular S_N_Ar followed by intramolecular McMurry condensation to provide the targets in a two‐step method (**Scheme** **4**). The synthesis began with the reaction of salicylaldehyde **7** derivatives, which reacted with 2‐fluorobenzaldehydes **6** to produces the diaryl ethers **5**. This SNAr reaction was performed under microwave irradiation at 120 °C for 30 min, using 3.33 equivalents of Cs_2_CO_3_ in DMSO under a nitrogen atmosphere. Under these conditions, the intermediate materials **5**, required for the next step, were obtained in yields of 73% (*R* ^1^ = *R* ^2^ = H), 80% (*R* ^1^ = F, *R* ^2^ = H), and 82% (*R* ^1^ = H, *R* ^2^ = OMe). Subsequently, these diaryl ethers **5** were applied to synthesize the dibenzo[*b*,*f*]oxepines **4** from intramolecular McMurry reaction using 3 equiv. of Zn and 1.5 equiv. of TiCl_4_ in THF at r.t. for 12 h. These products, bearing neutral, electron‐withdrawing (EWG), or electron‐donating groups (EDG), were obtained in comparable yields of 55%, 53%, and 55%, respectively (Scheme 4). The reactional mechanism occurs by dimerization of ketyl radicals affording the metallopinacol intermediate, subsequently yielding the desired compound **4** (Scheme 4).

**Scheme 5:**
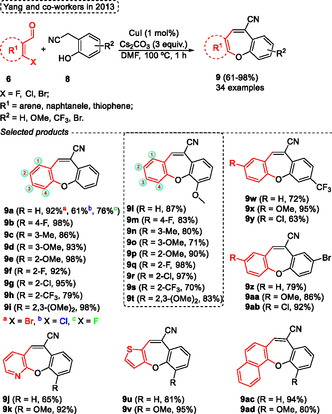
Synthesis of dibenzo[*b*,*f*]oxepine unit described by Yang and coworkers^[^ ^19^ ^]^ in 2013.

In the same year, Yang and coworkers^[^ ^19^ ^]^ reported the synthesis of numerous nitrile‐functionalized dibenzo[*b*,*f*]oxepine **9** in short reaction times (1 h) under a simple Cu‐assisted or Cu‐free condition by one‐pot cascade reaction using commercially available starting materials. The reactions of various 2‐(2‐hydroxyphenyl)‐acetonitriles **8** with a variety of 2‐haloarylaldehydes **6** were performed using CuI (1 mol%) as the catalyst, 3 equiv. of Cs_2_CO_3_ as the base, using DMF as solvent at 100 °C for 1 h. Under these conditions, novel highly functionalized dibenzo[*b*,*f*]oxepines **9** were synthesized (34 examples) in yields ranging from moderate to good (61%–98%) (**Scheme** **5**).

The protocol was not sensitive to different electron‐withdrawing or donating groups attached to both 2‐(2‐hydroxyphenyl)‐acetonitriles **8** and several 2‐bromoarylaldehydes **6** precursors. When the 2‐haloarylaldehydes **6** were evaluated, the effect of halogen variation on the yield of the target product **9a** was observed, starting from 2‐bromobenzaldehyde, 2‐chlorobenzaldehyde or 2‐fluorobenzaldehyde target product **9a** yields varied from 92%, 61% to 76%, respectively. Additionally, the method was extended to starting materials containing naphthyl or heteroaryl groups, such as 1‐bromonaphthalene‐2‐carbaldehyde, 2‐bromopyridine‐3‐carbaldehyde, or 3‐bromothiophene‐2‐carbaldehyde, in these cases, the target products **9j–k**, **9u–v**, **9ac–ad** were obtained with significantly satisfactory yields (65%–95%). The authors reported that, under the optimized conditions, the reaction in the absence of copper catalyst afforded the desired product **9a** in lower yield (60%) when compared to the use of 1 mol% of CuI as catalyst (92%). In this case, the starting material was not completely consumed (Scheme 5).

**Scheme 6:**
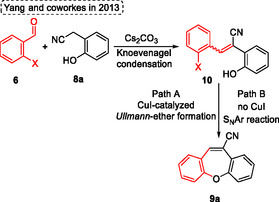
Synthesis of dibenzo[*b*,*f*]oxepine unit described by Yang and coworkers^[^ ^19^ ^]^ in 2013.

The proposed reactional mechanism was described by the authors, beginning with a Knoevenagel condensation between 2‐halobenzaldehydes **6** and 2‐(2‐hydroxyphenyl)‐acetonitriles **8a** to form intermediate 10. Subsequently, the target product **9a** can be formed by two pathways, in the presence of CuI the cyclization occurs by Ullmann ether formation (pathway A). In the absence of a copper catalyst, the reaction pathway follows an aromatic nucleophilic substitution mechanism (Path B) (**Scheme** **6**).

**Scheme 7:**
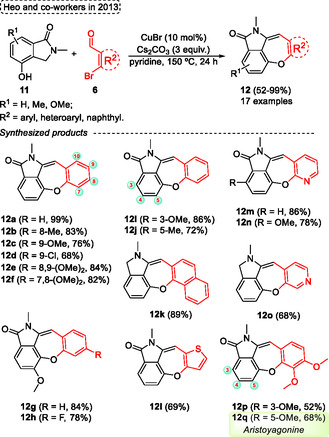
Synthesis of dibenzo[*b*,*f*]oxepine unit described by Heo and coworkers^[^ ^20^ ^]^ in 2013.

Based on the conditions previously reported by Yang (see Scheme 5), Heo and coworkers^[^ ^20^ ^]^ in the same year (2013) described a Cu‐catalyzed etherification/aldol condensation cascade reaction employing various 2‐bromobenzaldehydes **6** and 4‐hydroxyisoindolin‐1‐one **11** to synthesize the target dibenzoxepine lactams **12** (**Scheme** **7**). In this protocol, 2‐bromoarylaldehydes **6** were reacted with 4‐hydroxyisoindolin‐1‐ones **11** in the presence of CuBr (10 mol%) as the catalyst 3 equiv. of Cs_2_CO_3_ as the base in pyridine at 150 °C. After 24 h under these conditions, a broad range of desired dibenzoxepine lactams **12** (17 examples) was obtained in yields ranging from 52% to 99%. This protocol was efficiently applied to the total synthesis of the natural compound *Aristoyagonine* **12q**, in this case, the standard conditions were applied to afford the target of 68% yield. The eaction was not sensitive to electronic effects for both EDG or EWG attached in the aromatic ring of 2‐bromoarylaldehydes **6** precursors. Furthermore, 2‐bromoarylaldehydes containing heteroaromatic ring (such as pyridine, thiophene) and naphthyl substituents were successfully applied as starting materials, which afforded the compounds **12m**, **12n**, **12o**, **12l,** and **12k** in yields of 86%, 78%, 68%, 69%, and 89%, respectively (Scheme 7).

**Scheme 8:**
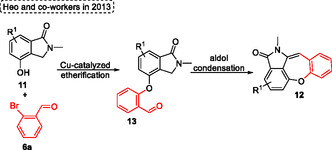
Synthesis of dibenzo[*b*,*f*]oxepine unit described by Heo and coworkers^[^ ^20^ ^]^ in 2013.

Interestingly, unlike the method developed by Yang, in this case the etherification occurs first, followed by an aldol condensation reaction, likely attributable to the varying acidities of the benzylic protons of 4‐hydroxyisoindolin‐1‐one and 2‐(2‐hydroxyphenyl)‐acetonitriles (See the key intermediates in the Scheme 6 and **8**).

**Scheme 9:**
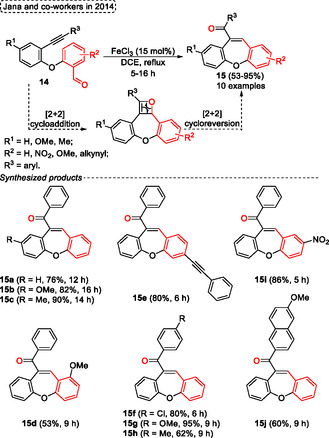
Synthesis of dibenzo[*b*,*f*]oxepine unit described by Jana and coworkers^[6c]^ in 2014.

A high regio‐ and chemoselective protocol using FeCl_3_ as a catalyst for the intramolecular‐metathesis reaction of alkynes with aldehydes unit of several 2‐(2′‐arylethynyl‐phenyloxy)‐benzaldehydes **14** for the synthesis of dibenzo[*b*,*f*]oxepines **15** and benzo[*b*]oxepines was reported by Jana and coworkers^[6c]^ in 2014 (**Scheme** **9**). This general synthetic offers several advantages, including the use of readily available starting materials, inexpensive catalyst, and atom‐economical procedure which makes it a powerful tool to obtain desired 7‐membered *O*‐heterocycles compounds **15**. The authors proposed a reaction pathway to obtain the target compounds, which probably occurs by [2 + 2] cycloaddition which the *σ*‐complex can active the carbonyl group or π‐complex active the alkyne unit, followed by cycloreversion in the subsequent step (Scheme 9).

In Jana's protocol, several 2‐(2′‐arylethynyl‐phenyloxy)‐benzaldehydes **14** were reacted in the presence of 15 mol% of FeCl_3_ catalyst in DCE under reflux. After 5–16 h under, a total of 10 examples of dibenzo[*b*,*f*]oxepines **15** were synthesized in yields ranging from moderate to excellent (53%–95%). The method was not sensitive to the electronic effect of substituent bonded in the aromatic ring of **14**. Functionalized substrates afforded the target compounds **15** in good yield, however, the steric effects were more sensitive, which the compound **15d** was obtained in relatively lower yield (53%) (Scheme 9).

**Scheme 10:**
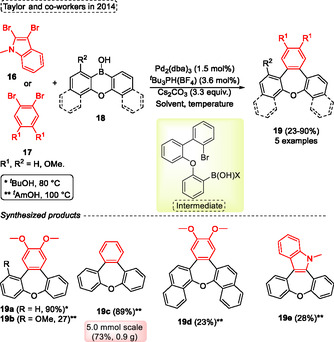
Synthesis of dibenzo[*b*,*f*]oxepine unit described by Taylor and coworkers^[^ ^21^ ^]^ in 2014.

In 2014 Taylor and coworkers^[^ ^21^ ^]^ reported the obtention of di‐ and tri‐benzo‐oxepines **19** compounds via a two‐fold Suzuki–Miyaura cross‐coupling reaction using cyclic diarylborinic acids **18,** as well as dihaloarenes **16** or **17** as starting material. Interestingly, the borinic acids **18,** the reagent utilized in this method exhibit relatively low reactivity, however, they were able to be used in two successive Suzuki–Miyaura cross‐coupling reactions, forming new two carbon–carbon bonds. In Taylor's protocol, cyclic diarylborinic acids **18** were reacted with dihaloarenes **16** or **17** in the presence of 1.5 mol% of Pd_2_(dba)_3_ as catalyst, 3.6 mol% of ^*t*^Bu_3_PH(BF_4_) as ligand, 3.3 equiv. of Cs_2_CO_3_ as a base. In general, the ^*t*^AmOH was employed as solvent at 100 °C, however when compound **19a** was synthesized, ^*t*^BuOH was used as solvent at 80 °C. Under these conditions, five examples of target compound **19** were obtained in yields ranging from low to excellent (23%–90%) (**Scheme** **10**).

In general, the authors performed the reaction on a 0.1 mmol scale. Notably, the synthesis of compound **19c** was scalable to a 5.0 mmol scale, showing only a slight decrease in the yield of the target compound **19c** (73%, 0.9 g). In addition, the substrates were sensitive to steric effects, the compounds **19b** and **19d** were obtained at only 27% and 23%, respectively. In some cases, side products were isolated, for example, the compound **19e** was isolated in 28% of yield with a side cross‐coupling/protodeboronation product. Similarly, it occurred in the formation of compound **19a**. The protocol was not efficient when 1,2‐dibromo alkenes were applied as starting material under standard conditions (Scheme 10).

In this context, due to the versatility and broad applicability of metal catalysts (especially Cu and Pd) in different synthetic protocols to obtain dibenzo[*b*,*f*]oxepines core, the use of Pd‐catalyzing an intramolecular C—H/C—Br coupling reaction to form fused perylene diimides with seven‐membered rings was described years later (2021).^[^ ^22^ ^]^

**Scheme 11:**
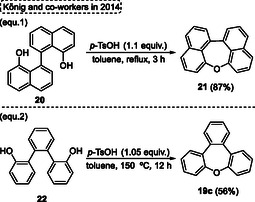
Synthesis of dibenzo[*b*,*f*]oxepine unit described by König and coworkers^[^ ^23^ ^]^ in 2014.

The first synthesis of highly condensed (naphthalene) oxepines **21** was reported by König and coworkers in 2014,^[^ ^23^ ^]^ to posteriorly study X‐ray crystallographic analysis, use of electrochemical analysis as well as theoretical calculations to obtain HOMO and LUMO energy levels data. In this context, the target [1,1′‐bisnaphthalene]‐8,8′‐diol **20** was prepared using conditions previously described in the literature from 1‐methoxynaphthalene by three successive reactions. The synthetic design for **20** starts with lithiation and oxidative carbon—carbon bond using Fe(acac)_3_, followed by demethylation using BBr_3_ to afford the desired target [1,1′‐binaphthalene]‐8,8′‐diol **20** in 54% overall yield. This starting material **20** was then cyclized in the presence of 1.1 equiv. of *p*‐toluenesulfonic acid (*p*‐TsOH) in toluene at reflux temperature for 3 h to obtain the target dinaphtho[1,8‐*bc*:1′,8′‐*ef*]oxepine **21** in good yield (87%) (**Scheme** **11**, eq. 1). Subsequently, the tribenzo[*b*,*d*,*f*]oxepine **19c** also was synthesized, adapting the conditions described above, in this case the [1,1′:2′,1″‐terphenyl]‐2,2″‐diol **22** was reacted in the presence of 1.05 equiv. of *p*‐TsOH in toluene at 150 °C for 12 h (Scheme 11, eq. 2).

**Scheme 12:**
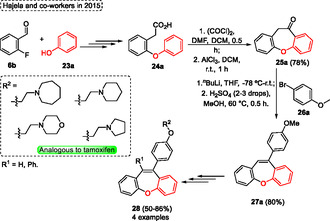
Synthesis of dibenzo[*b*,*f*]oxepine unit described by Hajela and coworkers^[^ ^24^ ^]^ in 2015.

Dibenzo[*b*,*f*]oxepines, **28** analogs to *tamoxifen*, were synthesized, and their activity for antiproliferative activity on breast cancer cell lines was checked by Hajela and coworkers in 2015 (**Scheme** **12**).^[^ ^24^ ^]^ This antibreast cancer agent was obtained from simple and commercially available starting materials, which starts the synthetic route by reacting 2‐fluoro‐benzaldehyde **6b** with phenol **23a** by nucleophilic substitution reaction. After some steps, the previously synthesized 2‐phenoxy phenylacetic acid **24a** was used as starting material in an intramolecular Friedel‐Crafts reaction, which was performed in the presence of oxalyl chloride, DMF as a catalyst, DCM for 0.5 h, followed by the addition of AlCl_3_ (Scheme 12).

The target dibenzo[*b*,*f*]oxepin‐10(11*H*)‐one **25a** was obtained in 78% yield after 1 h of reaction at r.t. Subsequently, to obtain the analogous the tamoxifen analog **28**, this cyclic ketone **25a** was reacted with 4‐methoxyphenylmagnesium bromide, however, the Grignard reaction did not work efficiently. In contrast, when the ketone was reacted with 4‐bromoanisole **26a** in the presence of butyllithium at −78 °C the carbinols were formed and directly transformed in the compounds **27a** (yield of 80%) by acid‐catalyzed dehydration step. Therefore, the tamoxifen analogs, (10‐(4‐ethoxyamino phenyl)dibenzo[*b*,*f*]oxepines **28**) were obtained *through several* steps starting from 10‐(4‐methoxyphenyl)dibenzo[*b*,*f*]oxepine **27a**, previously synthesized (Scheme 12).

**Scheme 13:**
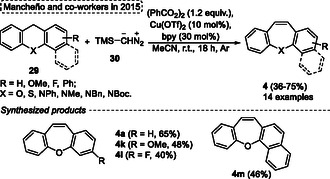
Synthesis of dibenzo[*b*,*f*]oxepine unit described by Mancheño and coworkers^[^ ^5^ ^]^ in 2015.

In 2015, Mancheño and coworkers^[^ ^5^ ^]^ reacted several xanthene or acridine **29** derivatives with TMSCHN_2_ **30** to promote the copper‐catalyzed Wagner–Meerwein‐type rearrangement to synthesize the tricyclic dibenzoxepines **4** and dibenzazepines (**Scheme** **13**). This work represents the first method of Cu‐catalyzed C—H bond functionalization followed by the ring expansion step. The authors react to several simple starting materials **29** in the presence of 10 mol% Cu(OTf)_2_ as a catalyst, 30 mol% of by as ligand, 1.2 equiv. of (PhCO_2_)_2_ in MeCN at r.t. for 18 h under an inert Ar atmosphere. Under these conditions, 14 examples **4** were synthesized under these conditions, which are obtained in moderated yields (36%–75%) (Scheme 13). The protocol was not sensitive to electronic and steric effects when different xanthenes **29** were evaluated under standard conditions. In these cases, both neutral (H atom), EDG (OMe), and EWG (F atom) are attached in the aromatic ring affording the target products **4a**, **4k**, and **4l** in 65%, 48%, and 40% yields, respectively. A similar result (46%) was obtained for the naphthyl fused product **4m** (Scheme 13).

**Scheme 14:**
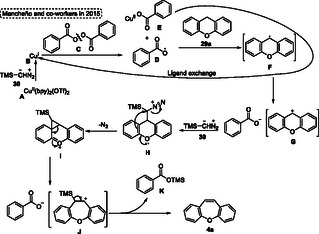
Mechanism proposed by Mancheño and coworkers^[^ ^5^ ^]^ in 2015.

The reaction pathway proposed by authors starts with the formation of active copper species **B** from the reduction of Cu(bpy)_2_ complex **A** by diazo alkane reagent **30**. Subsequently, the copper species mediates the homolytic O—O bond cleavage of peroxide species **C**, giving the radical intermediate **D** as well as the Cu^II^‐carboxylate species **E**. In the next step, radical intermediate **D** performs the two‐electron radical oxidation of the benzylic position, forming the carbocation intermediate **G**, which, after reaction, with diazo compound **30**, gives the intermediate **H**. Subsequently, this intermediate **H** suffers a nucleophilic attack of aromatic ring losing an N_2_ unit and forming the cyclopropyl intermediate **I**, which after a ring expansion reaction gives the 7‐membered carbocation intermediate **J**. The target product **4a** is formed after the TMS elimination step of **K** (**Scheme** **14**).

**Scheme 15:**
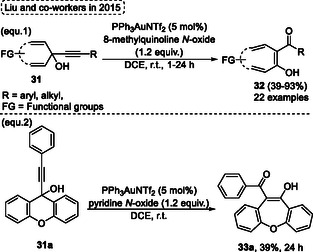
Synthesis of dibenzo[*b*,*f*]oxepine unit described by Liu and coworkers^[^ ^25^ ^]^ in 2015.

Another similar reaction was described by Liu and coworkers, in 2015,^[^ ^25^ ^]^ employing alkynyl quinols **31** substrates in oxidative ring expansion reactions using gold as a catalyst to obtain several substituted 7‐ or 6‐membered benzotropones, phenanthrenes, quinolin‐2(1H)‐ones **32** (**Scheme** **15**, eq. 1). This process uses 5 mol% of PPh_3_AuNTf_2_ and 1.2 equiv. 8‐methylquinoline *N*‐oxide in DCE at r.t. to give the compounds **32**, 22 examples, in yields ranging from moderated and excellent (39%–93%) in reaction times ranging from 1 to 24 h (Scheme 15, eq. 1). When starting from 9‐(phenylethynyl)‐9*H*‐xanthen‐9‐ol **31a** some changes in the reaction conditions were necessary, in this case the 8‐methylquinoline *N*‐oxide was changed by 1.2 equiv. pyridine *N*‐oxide. This change led to the formation of product **33a** in a lower yield of 39% after 24 h of reaction (Scheme 15, eq. 2).

**Scheme 16:**
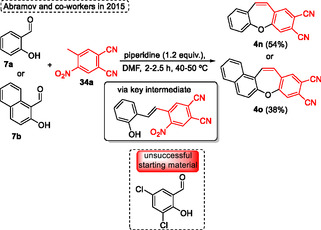
Synthesis of dibenzo[*b*,*f*]oxepine unit described by Abramov and coworkers^[^ ^26^ ^]^ in 2015.

In the following year, 2015, Abramov and coworkers^[^ ^26^ ^]^ reported a one‐pot, two‐step process involving a condensation followed by an SNAr reaction for the synthesis of dibenzo[*b*,*f*]oxepine‐2,3‐dicarbonitriles **4n** and benzo[*f*]naphtha‐[2,3‐*b*][1,4]oxazepine‐2,3‐dicarbonitrile **4o** (**Scheme** **16**). In this case, *o*‐hydroxyarylaldehydes **7** were efficiently applied as starting materials instead of *o‐halo benzaldehydes* **6** as frequently found in the literature (Scheme 16). The protocol reacts 4‐methyl‐5‐nitrophthalonitrile **34a** with *o*‐hydroxynaphthaldehyde **7b** or *o*‐hydroxybenzaldehyde **7a** in the presence of 1.2 equiv. of piperidine as a base in DMF as a solvent for reaction times ranging from 2 to 2.5 h at 40–50 °C. Under these conditions, the target dibenzo[*b*,*f*]oxepine‐2,3‐dicarbonitriles **4n** and **4o** were obtained in moderated yields of 54% and 38%, respectively, after the reaction of phenoxide ion performs the intramolecular substitution of the nitro group. The protocol was sensitive to EWG containing *o*‐hydroxybenzaldehydes, when chlorine atoms are attached in the aromatic ring the target product was not obtained (Scheme 16).

**Scheme 17:**
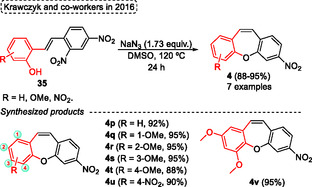
Synthesis of dibenzo[*b*,*f*]oxepine unit described by Krawczyk and coworkers^[^ ^27^ ^]^ in 2016.

Similarly to Abramov's protocol reported above, Krawczyk and coworkers^[^ ^27^ ^]^ in 2016 described the intramolecular nucleophilic substitution of 2‐hydroxy‐2′,4′‐dinitrostilbenes **35** using a simple and efficient protocol to synthesize the substituted dibenzo[*b*,*f*]oxepines using sodium azide (**Scheme** **17**). Since this scaffold is a key building block in medicinal chemistry due to its important biological activity, after the synthesis of target compounds, molecular docking was performed after the synthesis of the target compounds to investigate the tubulin protein interactions. In this method the intramolecular nucleophilic substitution of the hydroxy group is preferred over the substitution of the C2‐position nitro group by azide anion. The best conditions reported by the authors involved reacting 2‐hydroxy‐2′,4′‐dinitrostilbenes **35** in the presence of 1.73 equiv. of sodium azide, with DMSO as the solvent, 120 °C for 24 h (Scheme 17). Using these conditions the authors synthesized seven examples of target dibenzo[*b*,*f*]oxepine **4** in excellent yield (88%–95%). The protocol was not sensitive to electronic and steric effects when the starting materials **35** contained 4‐nitro or (1, 2, 3, or 4)‐methoxy groups under standard conditions. Only when the dibenzo[*b*,*f*]oxepine **4t** was synthesized a slight decrease in the yield was observed (**4t**, 88%), probably due to a not very pronounced steric effect (Scheme 17).

### 2.2 Protocols for Synthesizing Dibenzo[b,f]oxepine from 2017 to 2019

**Scheme 18:**
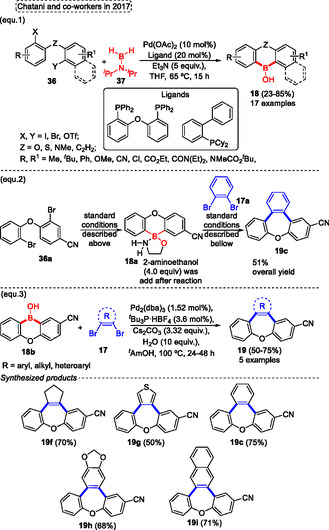
Synthesis of dibenzo[*b*,*f*]oxepine unit described by Chatani and coworkers^[^ ^28^ ^]^ in 2017.

Normally, the synthesis of cyclic diarylborinic acids requires organolithium reagents. In this regard Chatani and coworkers^[^ ^28^ ^]^ described, in 2017, the synthesis of cyclic diarylborinic acids **18** from the reaction of aryl **36** with the dihydroaminoborane reagent **37** (**Scheme** **18**, eq. 1). This protocol uses *N*, *N*‐diisopropylboranamine **37** as the boron source in the reaction with a wide range aryl halides **36** in the presence of 10 mol% of Pd(OAc)_2_ as a catalyst, 20 mol% of a ligand, 5 equiv. of Et_3_N in THF at 65 °C for 15 h, to obtain the target cyclic diarylborinic acids **18** (17 examples) with yields varying from low to high (23%–85%) (Scheme 18, eq. 1). The method was compatible with a range of functional groups, such as CN, CO_2_Et, CONEt_2,_ and NMeCO_2_^*t*^Bu. These compounds are of great interest in organic chemistry, biochemistry, as well as in materials science due to their low toxicity and because they are air‐ and moisture‐stable building blocks, which have great applicability as starting materials to obtain more complex compounds (Scheme 18, eq. 1).

The 10‐hydroxy‐10*H*‐dibenzo[*b*,*e*][1,4]oxaborinine‐2‐carbonitrile **18b** previously formed was used as a precursor in the reaction with different 1,2‐dibromo compounds **17** in the presence of 1.52 mol% of Pd_2_(dba)_3_ as catalyst, 3.6 mol% of ^*t*^Bu_3_PHBF_4_, 3.32 equiv. of Cs_2_CO_3_, 10 equiv. of H_2_O in ^*t*^AmOH as the solvent at 100 °C for 24–48 h. This annulation two‐fold Suzuki–Miyaura cross‐coupling reaction gives the target benzoxepines **19** (5 examples) in moderate yields (50%–75%). The versatility of this protocol was checked from different 1,2‐dibromo‐arenes **17**, which afforded the target tribenzo[*b*,*d*,*f*]oxepine **19h**, **19i**, and **19c** in yields of 68%, 71%, and 75%, respectively. Similar results were obtained when the 1,2‐dibromo‐alkyl compound **17** (1,2‐dibromo cyclopentene) was used as starting material, yielding the desired product **19f** in 70% of yield. Nevertheless, when the 1,2‐dibromo‐heteroarene (3,4‐dibromothiophene) was used under standard conditions desired dibenzo[*b*,*f*]thieno[3,4‐*d*]oxepine **19g** was obtained with a slightly reduced yield (50%) compared to the use of 1,2‐dibromo‐aryl or ‐alkyl starting materials (Scheme 18, eq. 3).

In addition, the protocol was extended by using 2‐aminoethanol in the first step, reacting with 3‐bromo‐4‐(2‐bromophenoxy)benzonitrile **36a**, the borinate intermediate **18a** was obtained after simple filtration and was used in the next step without further purification. This compound, **18a** was used as the starting material in a subsequent reaction with 1,2‐dribromobenzene **17a** to give the target tribenzo[*b*,*d*,*f*]oxepine **19c** in an overall of 51% (Scheme 18, eq. 2).

**Scheme 19:**
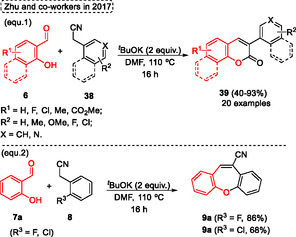
Synthesis of dibenzo[*b*,*f*]oxepine unit described by Zhu and coworkers^[^ ^29^ ^]^ in 2017.

The synthesis of a wide range of coumarins **39** was developed by Zhu and coworkers in 2017,^[^ ^29^ ^]^ in which a wide range of salicylaldehydes **6** were reacted in the presence of arylacetonitriles **38** to obtain the target *O*‐heterocycle **39** (20 examples) in yields ranging from moderate to excellent (40%–93%) (**Scheme** **19**, eq. 1). The starting materials **6** and **38** reacted only in the presence of 2 equiv. of ^*t*^BuOK as the base and DMF as the solvent at 110 °C for 16 h. The method tolerated several EWG and EDG, however, when the ortho halogen substituted arylacetonitriles **8**, such as 2‐(2‐fluorophenyl)acetonitrile and 2‐(2‐chlorophenyl) acetonitrile, were the precursors the dibenzo[*b*,*f*]oxepine‐10‐carbonitriles **9a** were isolated in 86% and 68% of yield, respectively, via an initial intermolecular condensation followed by intramolecular nucleophilic substitution (Scheme 19, eq. 2).

**Scheme 20:**
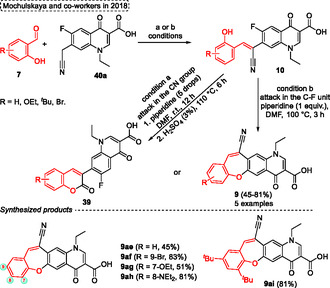
Synthesis of dibenzo[*b*,*f*]oxepine unit described by Mochulskaya and coworkers^[^ ^30^ ^]^ in 2018.

In 2018, the selective synthesis of novel (2‐oxo‐2‐*H*‐chromen‐3‐yl) substituted fluoroquinolones **39** or oxepine compounds **9** was developed by Mochulskaya and coworkers^[^ ^30^ ^]^ (**Scheme** **20**). The protocol reacts salicylic aldehydes **7** with 7‐(cyanomethyl)‐1‐ethyl‐6‐fluoro‐4‐oxo‐1,4‐dihydroquinoline‐3‐carboxylic acid **40a** to promote the condensation of aldehyde unit in the methylene carbon. Subsequently, this formed intermediate can react in two ways. Initially, the hydroxyl group can attack the CN group, resulting in the formation of (2‐oxo‐2‐*H*‐chromen‐3‐yl) substituted fluoroquinolones **39**. Alternatively, the hydroxyl group may react with the C‐F unit, leading to intramolecular S_N_Ar cyclization and yielding the desired oxepine **9** (Scheme 20). The structure of different products was determined by ^1^ H and ^13^C NMR spectroscopy, aided by 2D ^1^H–^1^H NOESY and ^1^H–^13^C HSQC, HMBC experiments. In this protocol, the amount of base, as well as the temperature used, directs the reaction path to the different products. To synthesize the compounds **9**, the intermediate **10** was reacted in the presence of five drops of piperidine in DMF at r.t. for 12 h followed by the addition of H_2_SO_4_ (3%) and an increase of temperature to 110 °C which remained for 6 h. In contrast, the OH group's attack in the C–F unit occurs in the presence of 1 equiv. piperidine using DMF as solvent at 100 °C for 3 h. Under these conditions, the target compounds **9** (5 examples) were isolated in yields ranging from moderate to good (45%–81%) (Scheme 20).

**Scheme 21:**
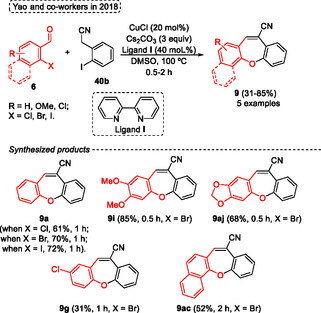
Synthesis of dibenzo[*b*,*f*]oxepine unit described by Yao and coworkers^[^ ^31^ ^]^ in 2018.

Interestingly, Yao and coworkers^[^ ^31^ ^]^ in the same year reported the reaction of aryl aldehydes with 2‐iodobenzylcyanides **40b** to give the 2‐aryl‐3‐cyanobenzofurans. However, when 2‐halobenzaldehyde **6** were reacted under similar conditions the dibenzo[*b*,*f*]oxepine‐10‐carbonitrile **9** were obtained (**Scheme** **21**). In this study, a similar protocol was developed to obtain the dibenzo[*b*,*f*]oxepine‐10‐carbonitrile **9** from the reaction of *o*‐haloaryl aldehydes **6** with *o*‐iodobenzylcyanides **40b** under a Cu‐catalyzed process. This reaction involves a Knoevenagel condensation that produces water in the reaction medium, which acts as an oxygen source to aryl hydroxylation followed by a Ullmann coupling reaction. The authors react different *o*‐haloarylaldehydes **6** with *o*‐iodobenzylcyanide **40b** in the presence of 20 mol% of CuCl as catalyst, 3 equiv. of Cs_2_CO_3_ as base, 40 mol% of ligand **I** in DMSO as solvent 100 °C. Under these conditions, 5 examples of **9** were obtained in generally good yields (52%–85%) in shorter reaction times (0.5–2 h), except for compound **9g**, which was obtained in lower yield (31%) (Scheme 21).

The authors evaluated the reactivity of halogen atoms bonded with 2‐halo benzaldehydes **6**. When the 2‐chloro, 2‐bromo, and 2‐ iodo benzaldehyde derivatives were reacted with **40** under standard conditions, the target dibenzo[*b*,*f*]oxepine‐10‐carbonitrile **9a** were obtained in yields of 61%, 70%, and 72%, respectively, after 1 h of reaction. A modest enhancement in product yield was observed as the size of the halogen atom increased, probably due to increased reactivity in the Ullmann coupling reaction (Scheme 21).

**Scheme 22:**
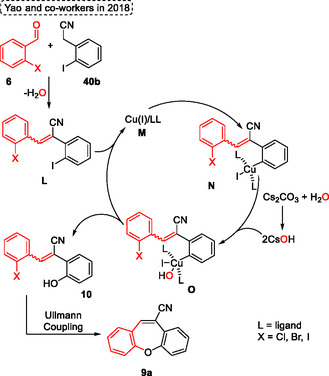
Mechanism proposed by Yao and coworkers^[^ ^31^ ^]^ in 2018.

The mechanism proposed by the authors starts with the Knoevenagel condensation reaction of 2‐haloarylaldehydes **6** with 2‐iodobenzylcyanide **40b** to give the intermediate **L**, accompanied by the release of a water molecule. This intermediate **L** then reacts with copper species **M** to yield another intermediate, **N**. Meanwhile, CsOH is generated from the reaction of Cs_2_CO_3_ with the water produced in the first step. The copper complex intermediate **N** reacts with CsOH to give the intermediate **O** via a nucleophilic addition. Subsequently, the copper **M** is regenerated to a new cycle, and the 3‐(2‐halophenyl)‐2‐(2‐hydroxyphenyl)acrylonitrile intermediate **10** is formed by the reductive elimination step. In the next step, this intermediate undergoes a Ullmann coupling yielding the target dibenzo[*b*,*f*]oxepine‐10‐carbonitrile **9a** (**Scheme** **22**).

**Scheme 23:**
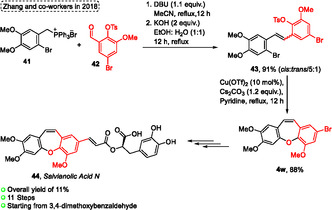
Synthesis of dibenzo[*b*,*f*]oxepine unit described by Zhang and coworkers^[^ ^32^ ^]^ in 2018.

The first total synthesis of *Salvianolic Acid N* **44** was described by Zhang and coworkers^[^ ^32^ ^]^ in 2018. This compound **44,** was synthesized with an overall yield of 11% after an 11 step reaction sequence starting from 3,4‐dimethoxybenzaldehyde commercially available. In this protocol, the Wittig reaction to obtain the *Z*‐stereoselectivity compound **43**, as well as the Ullmann intramolecular cyclization reaction to afford the 8‐bromo‐2,3,6‐trimethoxydibenzo[*b*,*f*]oxepine compound **4w,** are described by the authors as the key steps (**Scheme** **23**). The olefination reaction of ylide **41** with aldehyde **42** was optimized because the *trans*‐stilbene isomer was obtained preferentially under many conditions. The best conditions were established when the ylide **41** was reacted with aldehyde **42** in the presence of 1.1 equiv. of DBU, MeCN as solvent at reflux temperature for 12 h, followed by the addition of 2 equiv. of KOH in a 1:1 mixture of EtOH and H_2_O, also at reflux temperature for 12 h. Under these conditions, the target olefine **43** was isolated in excellent yield (91%) in an isomerization ratio of *cis*:*trans*/5:1 (Scheme 23). Subsequently, this compound **43** was used as the starting material in the intramolecular Ullmann reaction, in this case, some reactions using CuI as catalyst, K_2_CO_3_ as base, and DMF as solvent were performed, however, low yields (<26%) were obtained for compound **4w**. After some studies, the target 8‐bromo‐2,3,6‐trimethoxydibenzo[*b*,*f*]oxepine **4w** was obtained in excellent yield (88%) when the *trans*‐stilbene **43** was reacted in the presence of 10 mol% of Cu(OTf)_2_ as a catalyst, using 1.2 equiv. of Cs_2_CO_3_ as base and pyridine as solvent, at reflux temperature for 12 h. The presence of the bromine atom bonded to the aromatic ring is the key to the continuation of Pd‐catalyzed reactions to obtain the target *(+)‐Salvianolic Acid N* **44** (Scheme 23).

**Scheme 24:**
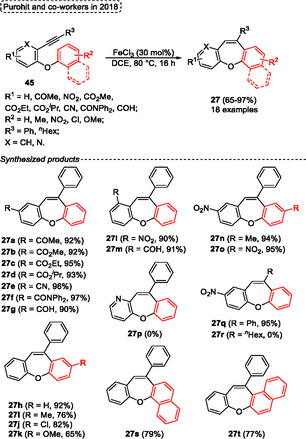
Synthesis of dibenzo[*b*,*f*]oxepine unit described by Purohit and coworkers^[^ ^33^ ^]^ in 2018.

In 2018, Purohit and coworkers^[^ ^33^ ^]^ reported the synthesis of dibenzo[*b*,*f*]oxepines **27** from the reaction of *o*‐phenoxy diarylacetylenes **45** by intramolecular C–H hydroarylation reaction (**Scheme** **24**). The authors employed FeCl_3_ as a catalyst, due to its high efficiency, low cost, and nontoxicity, which worked efficiently in the 7‐endo‐*dig* annulation of starting material **45**. After some studies, the best conditions were established when several *o*‐phenoxy diarylacetylenes **45** were reacted in the presence of 30 mol% of FeCl_3_ as catalyst, in DCE as solvent at 80 °C for 16 h. Under these conditions, a wide range (18 examples) of dibenzo[*b*,*f*]oxepines **27** were synthesized in yields ranging from moderate to good (65%–97%). The protocol was evaluated against several substituents bonded to both aromatic rings of ether moiety. In general, the protocol was sensitive to electronic effects, except for the compound **27n** (94%), the EWG groups attached to the aromatic ring afforded the target compounds in better yields when compared to EDG (See the Scheme 24). Moreover, the method did not work for starting materials **45** containing a pyridine unit as well as alkyl groups attached to the alkyne moiety. When 3‐phenoxy‐2‐(phenylethynyl)pyridine and 1‐(5‐nitro‐2‐phenoxyphenyl)oct‐1‐yn‐3‐one were reacted under standard conditions the target products **27p** and **27r** were not formed (Scheme 24).

**Scheme 25:**
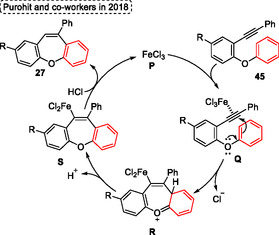
Mechanism proposed by Purohit and coworkers^[^ ^33^ ^]^ in 2018.

The mechanism (represented in **Scheme** **25**) was also proposed by the authors, and it starts with the interaction of FeCl_3_ **P** with the alkyne **45** unit to give the intermediate **Q**. Subsequently, this intermediate **Q** performs an intramolecular Friedel–Crafts type reaction to afford the cyclic intermediate **R** via 7‐endo‐*dig* annulation, after deprotonation by a chloride anion, gives the intermediate **S**. Finally, the HCl formed in the reaction medium reacts with intermediate **S** to generate the FeCl_3_ **P** catalyst for a new cycle, affording the target dibenzo[*b*,*f*]oxepine **27** (Scheme 25).

**Scheme 26:**
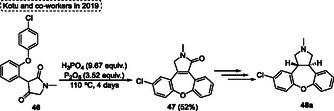
Synthesis of dibenzo[*b*,*f*]oxepine unit described by Kotu and coworkers^[^ ^34^ ^]^ in 2019.

In 2019, Kotu and coworkers^[^ ^34^ ^]^ reported a multistep, scalable method for the synthesis of several fused lactam **47** (up to 10 kg batch scale), as well as the isomerization of *cis*‐lactam to target *trans*‐lactam **48a**. In this regard, the *cis*‐isomer of compound **48a** can be achieved from an intramolecular reaction of 3‐(2‐(4‐chlorophenoxy)phenyl)‐1‐methylpyrrolidine‐2,4‐dione **46** using 9.67 equiv. of H_3_PO_4_ and 3.52 equiv. of P_2_O_5_ at 110 °C for 4 days. Under these conditions, the target compound **47** was obtained in 52% yield (**Scheme** **26**).

**Scheme 27:**
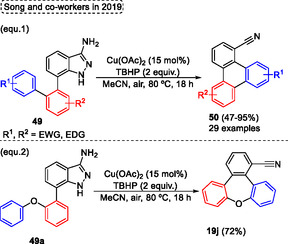
Synthesis of dibenzo[*b*,*f*]oxepine unit described by Song and coworkers,^[^ ^35^ ^]^ in 2019.

In 2019, 3‐aminoindazoles **49** were efficiently applied to obtain nitrile‐containing triphenylenes **50** by Song and coworkers,^[^ ^35^ ^]^ forming the cyano group in situ by radical reaction. This Cu‐catalyzed protocol enabled the synthesis of a wide range of triphenylenes **50** (29 examples) in yields ranging from moderate to good (47%–95%) (**Scheme** **27**, eq. 1). These compounds **50** were synthesized by oxidative denitrogenation of several 3‐aminoindazoles **49** in the presence of 15 mol% of Cu(OAc)_2_, 2 equiv. of TBHP in MeCN under an air atmosphere at 80 °C for 18 h. Additionally, the protocol was extended when the 7‐bisaryl unit of starting material **50** was changed by a unit containing heteroatom ones. In this regard the 7‐(2‐phenoxyphenyl)‐1*H*‐indazol‐3‐amine **49a** was efficiently applied under the standard conditions described above, giving the desired tribenzo[*b*,*d*,*f*]oxepine‐1‐carbonitrile **19j** in good yield (72%) (Scheme 27, eq. 2).

**Scheme 28:**
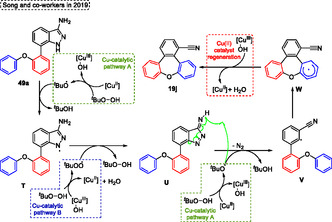
Mechanism proposed by Song and coworkers,^[^ ^35^ ^]^ in 2019.

The authors conducted control experiments to evaluate the mechanism, such as radical trapping using 2,2,6,6‐tetramethyl‐1‐piperidinyloxy (TEMPO) and 2,6‐ditert‐butyl‐4‐methylphenol (BHT), in both cases the desired product **19j** was not formed, these results indicate that reaction occurs by a radical mechanism. The mechanism proposed by authors starts with the formation of ^*t*^butoxyl radical from the reaction of Cu(II) species with TBHP by one‐electron transfer, posteriorly the ^*t*^BuO radical formed react abstracting hydrogen of NH bond of starting material, to afford ^*t*^BuOH and the radical intermediate **T**. In the next step the ^*t*^BuOO• radical, previously formed, reacts with intermediate **T**, performs a new proton abstraction, to give the intermediate **U**, which in the presence of another ^*t*^BuO• radical species afforded the nitrile intermediate **V** by dinitrogen reaction. Finally, the intermediate **V** suffers a 7‐endo‐*trig* radical cyclization step to give the intermediate **W**, which afforded the target compound **19j** after the oxidation step (**Scheme** **28**).

The copper catalyst performs four steps in the reaction process, starting with the reaction of TBHP with Cu(II) species to produce ^*t*^BuO• radicals and generate the Cu(III)–OH species (Cu‐catalytic pathway A). Posteriorly, the Cu(III)–OH species previously formed can react with another TBHP equivalent, generating ^*t*^BuOO• radicals and regenerate the Cu(II) species (Cu‐catalytic pathway B). Next, occurs another Cu‐catalytic pathway A. Finally, the fourth step occurs, which the Cu(II) catalyst is regenerated to a new cycle by reduction step of Cu(III)–OH species [Cu(II) catalyst regeneration]. This redox cycling between Cu(II) and Cu(III) species, which continuously generates ^*t*^BuO• and ^*t*^BuOO• radicals, is a key aspect of the copper‐catalyzed system, enabling the transformation and sustaining the catalytic turnover (Scheme 28).

**Scheme 29:**
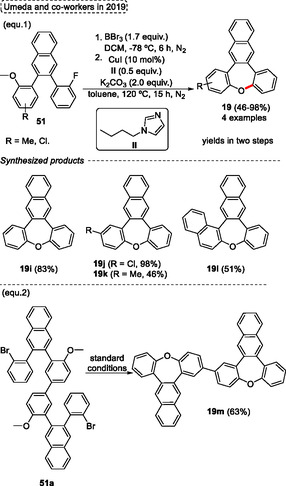
Synthesis of dibenzo[*b*,*f*]oxepine unit described by Umeda and coworkers^[^ ^36^ ^]^ 2019.

In 2019, Umeda and coworkers^[^ ^36^ ^]^ focused their attention on the synthesis of *π*‐extended oxepines **19** from the facile and practical protocol as well as, the study of optical properties. The synthesis of target oxepine **19** was performed by demethylation of the anisyl unit, followed by Cu‐catalyzed intramolecular cyclization of the starting materials **51** (**Scheme** **29**, eq. 1). These compounds **51** were previously synthesized by the reaction of *o*‐iodoanisole with 1‐bromo‐2‐ethynylbenzene via Sonogashira cross‐coupling followed by intramolecular cyclization. The 2‐(2‐fluorophenyl)‐3‐(2‐methoxyphenyl)naphthalenes **51** were reacted in a two‐step protocol. The first step occurs in the presence of 1.7 equiv. of BBr_3_, in DCM at −78 °C for 6 h under a N_2_ atmosphere. In the second step, 10 mol% of CuI, 0.5 equiv. of **II**, and 2.0 equiv. of K_2_CO_3_ were used in toluene at 120 °C for 15 h, also under N_2_ atmosphere. Under these conditions four examples of *π*‐extended oxepines **19** were obtained in yields varying from moderate to excellent (46%–98%, yields in two steps) (Scheme 29, eq. 1). The protocol was sensitive to both electronic and steric effect. For starting materials **51** containing neutral or EWG attached in the aromatic ring the yield of target products **19i** and **19j** (83% and 98%, respectively) were higher compared to those containing EDG ones, which the product **19k** was obtained in 46% of yield. A similar result was observed when the more sterically hindered starting material was used, in this case, the desired product **19l** was isolated in 51% of yield. Still, the protocol was efficiently applied to a more complex compound (Scheme 29, eq. 2), in this case, two successive cyclization reactions were performed using the standard conditions, giving the highly *π*‐extended target compound **19m** in 63% of yield (Scheme 29, eq. 2).

**Scheme 30:**
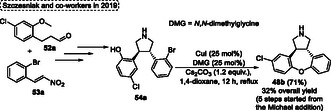
Synthesis of (+)‐asenapine **48b** described by Szcześniak and coworkers^[^ ^37^ ^]^ in 2019.

The (+)‐asenapine **48b** is the active ingredient in Saphris (USA), and Sycrest (Europe). These drugs are used for the treatment of schizophrenia and acute mania associated with bipolar disorder. Due to the increased use of Saphris, research into the synthesis of these compounds is of great interest. Based on this, Szcześniak and coworkers in 2019,^[^ ^37^ ^]^ described the total synthesis of (+)‐asenapine **48b** in five steps with high enantiomeric excess(ee) (>94% ee) (**Scheme** **30**).

The route to obtain the target (+)‐asenapine **48b** starts with the reaction of aldehydes **52a** with *trans*‐nitroalkenes **53a** via organocatalytic Michael addition reaction, followed by a reductive cyclization step and the protection and deprotection of nitrogen and oxygen atoms, which afforded the pyrrole **54a** (Scheme 30). This compound **54a** was reacted with 25 mol% of *N*,*N*‐dimethylglycine (DMG), 25 mol% of CuI as the catalyst, 1.2 equiv. of Cs_2_CO_3_ as the base, in 1,4‐dioxane. The mixture was reacted for 12 h at reflux temperature, under the conditions described above, the target product (+)‐asenapine **48b** was formed in a yield of 71% as a single *trans‐*isomer, via an intramolecular Ullmann reaction (Scheme 30).

### 2.3 Protocols for Synthesizing Dibenzo[*b*,*f*]oxepine from 2020 to 2022

**Scheme 31:**
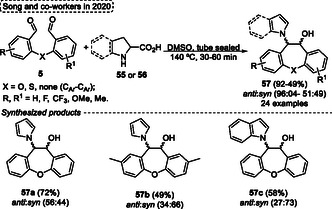
Synthesis 10,11‐dihydrodibenzo[*b*,*f*] oxepin‐10‐ols **57** described by Song and coworkers^[^ ^38^ ^]^ in 2020.

The synthesis of 9,10‐dihydrophenanthren‐9‐ol **57**, substituted with pyrrolyl or indolyl unit, was reported by Song and coworkers in 2020 (**Scheme** **31**).^[^ ^38^ ^]^ In this novel approach, an intramolecular [3 + 2] cycloaddition followed by ring‐opening aromatization occurs efficiently to give the target compounds **57**. The ideal conditions were found by the authors when they reacted the dialdehyde **5** with *L*‐proline **55** or indoline‐2‐carboxylic acid **56** in the presence of DMSO in a sealed tube, at 140 °C, for short reaction times (30–60 min) (Scheme 31). Under these conditions, several 9,10‐dihydrophenanthren‐9‐ol **57** were synthesized in good yields. When 2,20‐oxydibenzaldehydes **5** were reacted with *L*‐proline **55** or indoline‐2‐carboxylic acid **56** under standard conditions the 10,11‐dihydrodibenzo[*b*,*f*] oxepin‐10‐ols **57a**, **57b,** and **57c** were formed in yields of (selectivity *anti*:*syn*) 72% (56:44), 49% (34:66) and 58% (27:73), respectively (Scheme 31).

**Scheme 32:**
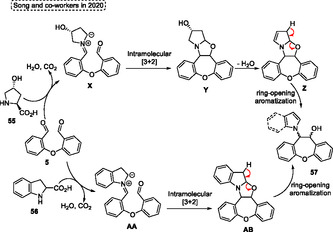
Mechanism proposed by Song and coworkers^[^ ^38^ ^]^ in 2020.

The authors proposed a plausible reaction pathway to the obtaining of target 10,11‐dihydrodibenzo[*b*,*f*] oxepin‐10‐ols **57**, which starts with the formation of an azomethineylide intermediate **X** or **AA** after reaction of **5** with **55** or **56**. Subsequently, these intermediates, **X** or **AA** undergo an intramolecular [3 + 2] cycloaddition to obtain the bicyclic 1,3‐oxazolidine intermediates **Y** or **AB**. Next, the dehydration step of the alcohol intermediate **Y** leads to the intermediate **Z**. Finally, the target compounds **57** are obtained after the ring‐opening aromatization step of intermediates **Z** or **AB** (**Scheme** **32**).^[^ ^38^ ^]^

**Scheme 33:**
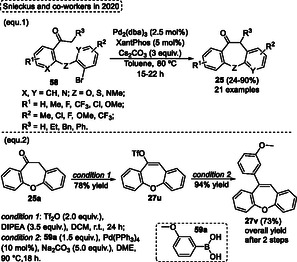
Synthesis dibenzoxepinone **25** and arylated dibenzo[*b*,*f*]oxepine **27v** described by Snieckus and coworkers^[^ ^39^ ^]^ in 2020.

The palladium‐catalyzed synthesis of Manske's ketone **25** by *α*‐arylation reaction was described by Snieckus and coworkers in 2020 (**Scheme** **33**).^[^ ^39^ ^]^ Widely substituted dibenzoxepinones **25** were synthesized in this protocol when diaryl ethers **58** were reacted in the presence of a catalyst system composed of Pd_2_(dba)_3_/XantPhos (2.5 mol%/5 mol%, respectively) in the presence of 3 equiv. of Cs_2_CO_3_ as the base, with toluene as solvent. These reactions were performed at 80 °C for 15–22 h, and several Manske's ketones **25** were obtained (21 examples) in yields ranging from low to excellent (24%–90%) (Scheme 33, eq. 1).

Additionally, the authors applied the dibenzoxepinone **25a** previously synthesized as a starting material in the synthesis of 10‐(3‐methoxyphenyl)dibenzo[*b*,*f*]oxepine **27v** by sequential triflation and Suzuki–Miyaura coupling reaction steps, as represented in Scheme 33, eq. 2. The intermediate **27u** was formed in 78% after the triflation reaction, and the Suzuki–Miyaura coupling step afforded the target arylated dibenzo[*b*,*f*]oxepine **27v** in 94% yield. The overall yield for the synthesis of the desired compound **27v** was 73% after 2 steps (Scheme 33, eq. 2).^[^ ^39^ ^]^

**Scheme 34:**
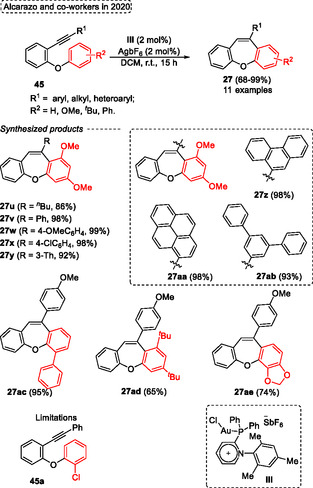
Synthesis of dibenzo[*b*,*f*]oxepine unit described by Alcarazo and coworkers^[^ ^40^ ^]^ in 2020.

Based on the stability of organic compounds, it is not surprising that the 6‐exo‐*dig* cyclization reaction is favored compared to 7‐endo‐*dig* ones, since these typically require more energy and lead to relatively higher activation enthalpies. In this sense, a protocol using Au(I)‐catalysis to promote the 7‐endo‐*dig* cyclization reaction by regioselective protonation of the triple bond of starting materials 1‐(benzyl‐, oxyaryl‐, thioaryl‐, and silaaryl)‐2‐ethynylbenzenes was established by Alcarazo and coworkers in 2020 (**Scheme** **34**).^[^ ^40^ ^]^ The 7‐membered (hetero)cycles dibenzo‐cycloheptatrienes, ‐oxepines, ‐thiepines, and ‐silepines were synthesized from the reaction of the respective starting materials in the presence of 2 mol% of Au‐catalyst **III** and 2 mol% of AgBF_6_ in DCM as the solvent at r.t. for 15 h. When *o*‐phenoxy diarylacetylenes **45** were employed under the conditions described above, 11 examples of dibenzo[*b*,*f*]oxepines **27** were obtained in yields ranging from good to excellent (68%–99%). Overall, the method efficiently tolerated substrates substituted in the alkyne unit with alkyl (^*n*^Bu), heteroaryl (3‐Th), and aryl containing neutral (Ph), EDG (4‐OMeC_6_H_4_) and EWG (4‐ClC_6_H_4_) groups. In the aforementioned situations, the products **27u** (*R* = ^*n*^Bu), **27y** (*R* = 3‐Th), **27v** (*R* = Ph), **27w** (*R* = 4‐OMeC_6_H_4_), and **27x** (*R* = 4‐ClC_6_H_4_) were isolated in 86%, 92%, 98%, 99%, 98%, respectively (Scheme 34). The protocol was also efficiently extended to alkynes substituted with different polyaryl groups, such as 1‐pyrene, 5′‐(1,1′:3′,1″‐terphenyl), and 9‐phenanthrene, affording the target dibenzo[*b*,*f*]oxepines **27aa**, **27ab,** and **27z** in excellent yields, 98%, 93%, and 98%, respectively. Unfortunately, the protocol did not work when the 1‐chloro‐2‐(2‐(phenylethynyl)phenoxy)benzene **45a** was applied under standard conditions. The authors report that substituents on the terminal portion of the alkyne do not influence the reaction. In contrast, the aromatic ring that undergoes the Friedel–Craft alkenylation reaction requires that it be neutral or substituted with an EDG. In this regard, when the starting material containing the EWG group attached to the aromatic ring was tested, the reaction did not work (Scheme 34).

**Scheme 35:**
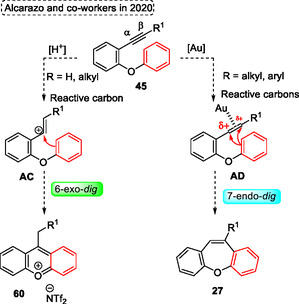
Mechanism proposed by Alcarazo and coworkers^[^ ^40^ ^]^ in 2020.

Analyzing the reactivity of the starting material when reacted with an Au‐catalyst, it is possible to verify that both alkyne carbons can be activated by this catalyst. However, due to the *α* position being sterically more accessible, the arene attack occurs at this position, exclusively delivering the desired dibenzoxepine **27**. Alternatively, when the starting material **45** was applied to the reaction using Brønsted acid catalysis, the two possible products were reported by the authors. This secondary product can be obtained by the formation of the most stable vinyl carbocation intermediate **AC**, from the regioselective protonation of the alkyne unit (**Scheme** **35**).

**Scheme 36:**
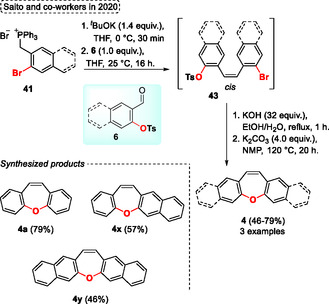
Synthesis of dibenzo[*b*,*f*]oxepine unit described by Saito and coworkers^[8b]^ in 2020.

Saito and coworkers in 2020,^[8b]^ studied the lowest singlet excited‐state (S1) energy profile of different *π*‐expanded oxepins **4**, which is directly related to the curved‐to‐planar conformational change in S1 [excited state aromaticity]. To carry out this experiment, the authors applied the synthesized compounds to the time‐resolved fluorescence spectroscopy technique. Firstly, to obtain the desired *π*‐expanded oxepins **4**, different bromoaryl phosphonium ylides **41** were reacted in the presence of 1.4 equiv. of ^*t*^BuOK using THF as the solvent at 0 °C for 30 min, followed by the addition of 1.0 equiv. of tosyl substituted aryl aldehydes **6** (**Scheme** **36**). This mixture remained for 16 h at 25 °C to obtain the *cis*‐olefin product **43** derivative of the Wittig reaction, which was extracted with EtOAc and used in the next step without additional purification procedure. Subsequently, the *cis*‐olefin **43** containing the tosyl group was reacted in the presence of 32 equiv. of KOH using a mixture of solvents EtOH/H_2_O in a ratio of (1:1) under reflux temperature for 1 h to carry out the deprotection of the tosyl group. Finally, the nucleophilic aromatic substitution reaction was performed using 4.0 equiv. of K_2_CO_3_, with NMP as the solvent at 120 °C for 20 h. Under these conditions the target dibenzo[*b*,*f*]oxepine **4a**, benzo[*b*]naphtho[2,3‐*f*]oxepine **4x**, and dinaphtho[2,3‐*b*:2′,3′‐*f*]oxepine **4y** were obtained in an overall yield of 79%, 57%, and 46%, respectively (yield based on the amount of aldehyde **6** used in the first step) (Scheme 36).

**Scheme 37:**
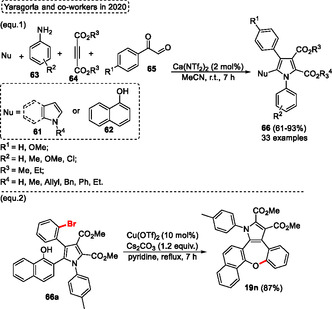
Synthesis of dibenzo[*b*,*f*]oxepine unit described by Yaragorla and coworkers^[^ ^41^ ^]^ in 2020.

The four‐component cyclization reaction catalyzed by calcium(II) to obtain fully substituted pyrroles **66** was described by Yaragorla and coworkers in 2020 (**Scheme** **37**, eq. 1).^[^ ^41^ ^]^ In this high‐atom‐economy protocol, readily available starting materials were used, including *α*‐keto aldehydes **65**, anilines **63**, activated alkynes **64**, and aromatic nucleophiles **61** or **62** (such as indole, pyrrole and naphthol), which were reacted in the presence of 2 mol% of Ca(NTf_2_)_2_, with MeCN as the solvent at r.t. for 7 h. The protocol forms acylimines as intermediates, which undergo successive reactions to afford the target penta‐substituted pyrroles **66** (33 examples) in yields ranging from moderate to excellent (61%–93%). The compound **66a**, previously synthesized, was used as starting material in an intramolecular *O*‐arylation reaction using a copper catalyst [Cu(OTf)_2_ (10 mol%)], in the presence of 1.2 equiv. of Cs_2_CO_3_ as a base in pyridine at reflux temperature. After 7 h of reaction, the target complex structure oxepine compound **19n** was obtained in good yield (87%) (Scheme 37, eq. 2).

**Scheme 38:**
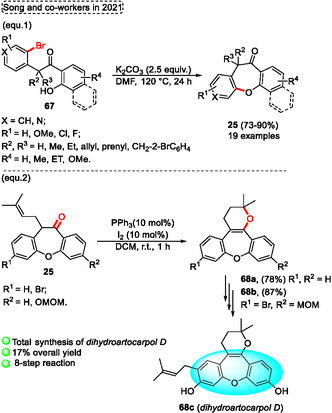
Synthesis of dibenzo[*b*,*f*]oxepine unit described by Song and coworkers^[^ ^42^ ^]^ in 2021.

A metal‐free protocol to obtain dibenzo[*b*,*f*]oxepinones **25** from an intramolecular *O*‐arylation reaction using 2‐halo benzyl 2‐hydroxyphenyl ketones **67** as starting materials was reported by Song and coworkers in 2021 (**Scheme** **38**, eq. 1).^[^ ^42^ ^]^ A wide range of 2‐halo benzyl 2‐hydroxyphenyl ketones **67** was efficiently reacted in the presence of 2.5 equiv. of K_2_CO_3_ as a base in DMF at 120 °C for 24 h to obtain the target dibenzo[*b*,*f*]oxepinones **25** (19 examples) in yields ranging from good to excellent (73%–90%). This protocol was applied as a key step in the total synthesis of *dihydroartocarpol D* **68c**. In this instance, the dibenzo[*b*,*f*]oxepinone, previously synthesized using the conditions described above, was reacted in the presence of 10 mol% of PPh_3_, 10 mol% of I_2_ in DCM at r.t. for 1 h. Under these conditions the dihydrodibenzo[*b*,*f*]pyrano[2,3‐*d*]oxepine **68a** was synthesized in a 78% yield. This compound **68a** was applied to the total synthesis of *dihydroartocarpol* D **68c**, which was obtained in 17% overall yield after an 8‐step reaction (Scheme 38, eq. 2).

**Scheme 39:**
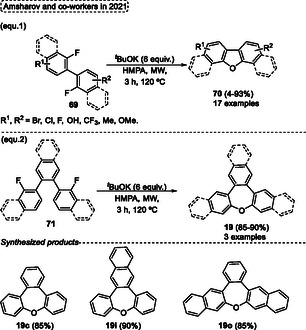
Synthesis of dibenzo[*b*,*f*]oxepine unit described by Amsharov and coworkers^[^ ^43^ ^]^ in 2021.

The synthesis of oxygen‐containing heterocycles **70** from starting materials without the presence of oxygen **69**, is rarely reported. In this regard, the reaction of 2,2′‐difluorinated biaryl **69** with potassium ^*t*^butoxide as an oxygen source was described by Amsharov and coworkers in 2021.^[^ ^43^ ^]^ The oxygen synthon is generated through the use of KO^*t*^Bu in the “ladderization” of fluorinated oligophenylenes (LooP) reaction to obtain *O*‐heteroarenes (**Scheme** **39**, eq. 1). When 2,2′‐difluorinated biphenyls **69** were reacted in the presence of 6 equiv. ^*t*^BuOK, HMPA, using microwave irradiation (MW) as an alternative energy source for 3 h at 120 °C a wide range of dibenzofuran substituted **70** (17 examples) were obtained in yields ranging from low to excellent (4%–93%). The target seven‐membered rings *O*‐heterocycles **19** were also synthesized. In these cases, the 2,2″‐difluoro‐1,1′:2′,1″‐teraryl compounds **71** were used as starting materials under standard conditions described in the Scheme 39, eq. (1). Gratifyingly, three highly *π*‐extended oxepines **19c**, **19i**, and **19o**, were obtained in excellent yields (85%–90%) (Scheme 39, eq. 2).

**Scheme 40:**
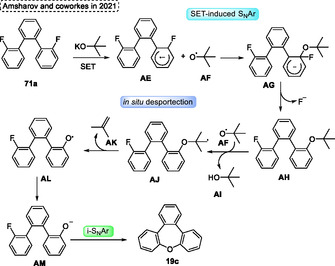
Mechanism proposed by Amsharov and coworkers^[^ ^43^ ^]^ in 2021.

The LooP mechanism to obtain the target *π*‐extended oxepines **19** starts with the reaction of starting material **71a** with ^*t*^BuOK by single‐electron transfer (SET) to afford the radical anion intermediate **AE** and ^*t*^BuO· radical **AF**. Subsequently, the intermediate **AG** is formed after SET‐induced S_N_Ar, which loses a fluorine atom giving the ether intermediate **AH**. In the next step, the ^*t*^BuO· radical **AF** abstracts a proton of **AH** yielding the methyl radical intermediate **AJ**, which undergoes an in situ deprotection step forms the 2‐methylprop‐1‐ene **AK**, as well as the oxygen radical, centered **AL**. The radical intermediate **AL** forms the anion intermediate **AM**. This previously generated intermediate then undergoes an intramolecular nucleophilic aromatic substitution to give the target oxepine **19c** (**Scheme** **40**).

**Scheme 41:**
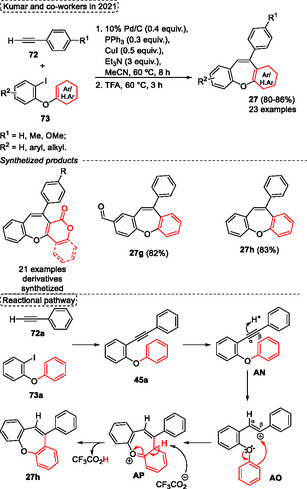
Synthesis and mechanism of dibenzo[*b*,*f*]oxepine unit described by Kumar and coworkers^[^ ^44^ ^]^ in 2021.

Kumar and coworkers in 2021,^[^ ^44^ ^]^ described the Pd/C and TFA‐promoted the direct synthesis of dibenz[*b*,*f*]oxepines **27** from iodoheteroaryl/aryl ethers **73** and terminal alkynes **72** under a highly efficient one‐pot method (**Scheme** **41**). This simple operational telescoping protocol offers the advantage of obviating the necessity to isolate the intermediate 2‐phenoxy diaryl acetylenes **45**, as illustrated in Scheme 24, along with its accessible starting materials and broad substrate scope. This reaction starts with the intermolecular C—C bond formation between aryl iodides **73** and terminal alkynes **72**, followed by intramolecular hydroarylation at the alkyne portion, yielding the desired dibenzo[*b*,*f*]oxepines **27** and benzo[6,7] oxepino[3,2‐*c*]chromen‐6‐ones (Scheme 41).

A wide range of iodoheteroaryl/aryl ethers **73** was reacted with terminal alkynes **72** to synthesize 21 examples of benzo[6,7] oxepino[3,2‐*c*]chromen‐6‐ones **27** in good yields (80%–86%). This method employs a two‐step protocol. Firstly, it uses 0.4 equiv. of 10% Pd/C as a catalyst, 0.3 equiv. of PPh_3_, 0.5 equiv. of CuI, and 3 equiv. of Et_3_N, in the presence of MeCN as the solvent at 60 °C for 8 h. Subsequently, 2 mL of TFA was added to the reaction medium, and the mixture was reacted at 60 °C for 3 h. Under these conditions (two examples of dibenz[*b*,*f*]oxepines) the 10‐phenyldibenzo[*b*,*f*]oxepine‐2‐carbaldehyde **27g** and 10‐phenyldibenzo[*b*,*f*]oxepine **27h** were synthesized in 82% and 83%, respectively (Scheme 41). Nevertheless, the authors proposed a plausible reaction pathway, which starts with the coupling of iodoheteroaryl/aryl ethers **73a** with a terminal acetylene **72a** using the Pd/C catalyst system to give the 2‐phenoxy diaryl acetylenes intermediate **45a**. Next, the intermediate **AF** is formed after acidic species activate the alkyne group. Subsequently, the intramolecular cyclization reaction in the **AO** intermediate afforded the nonaromatic seven‐membered ring **AP**, which yields the target compound **27h** after double aromatization of **AP** by the trifluoroacetate anion, which regenerates the TFA (Scheme 41).

**Scheme 42:**
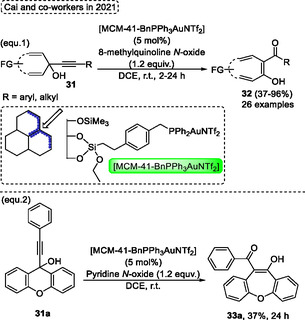
Synthesis of dibenzo[*b*,*f*]oxepine unit described by Cai and coworkers^[^ ^45^ ^]^ in 2021.

On account of the high interest in new heterogeneous catalysts, especially the mesoporous silica MCM‐41 materials, which possess unique properties previously described in the literature, in 2021, Cai and coworkers^[^ ^45^ ^]^ reported the oxidative ring expansion of alkynyl quinols **31** to obtain the different 6‐ and 7‐membered compounds **32** such as dibenzotropones, phenanthrenes, and quinolin‐2(1*H*)‐ones derivatives, via a heterogeneous gold(I) catalyst (**Scheme** **42**, eq. 1), similar to the protocol described by Liu and coworkers^[^ ^25^ ^]^ in 2015 (Scheme 15). Several tropone **32** (26 examples) were obtained in yields ranging from low to excellent (37%–96%) when a wide range of alkynyl quinols **31** was reacted in the presence of 5 mol% of [MCM‐41‐BnPPh_3_AuNTf_2_], 1.2 equiv. of 8‐methylquinoline *N*‐oxide as an oxidant, with DCE as the solvent at r.t. for reaction times ranging from 2 h to 24 h. The recovery efficiency of the heterogeneous gold(I) complex was performed and there is no difference in reactivity even after eight times recycling, which was recovered after a simple filtration step. In this context, when the 9*H*‐xanthen‐9‐one **31a** was applied under conditions similar to those described above, with the only modification being the oxidant species from 8‐methylquinoline *N*‐oxide to pyridine *N*‐oxide, the designated oxepine **33a** was obtained in only 37% yield (Scheme 42, eq. 2).

**Scheme 43:**
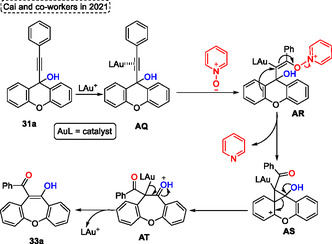
Mechanism proposed by Cai and coworkers^[^ ^45^ ^]^ in 2021.

The proposed mechanism starts with the formation of the intermediate **AQ** by coordination of Au from the heterogeneous catalyst with an alkyne unit. Subsequently, the reaction of intermediate **AQ** with pyridine *N*‐oxide gives the intermediate **AR**, which after performing the nucleophilic attack of the double bond of the aromatic ring in the vinyl gold portion affords the cyclopropylcarbinyl cation intermediate **AS**. In the subsequent step, the intermediate **AS** suffers a ring‐opening reaction followed by ring expansion to give the intermediate **AT**. Finally, the target benzoxepine **33a** is formed (**Scheme** **43**).

**Scheme 44:**
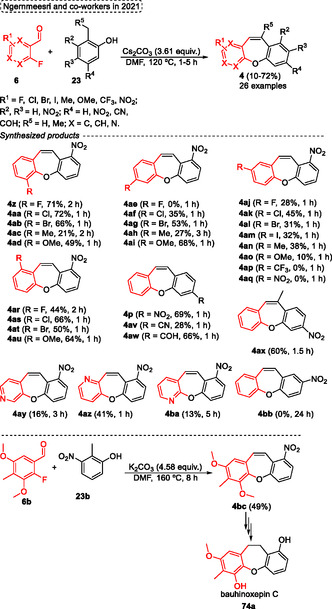
Synthesis of dibenzo[*b*,*f*]oxepine unit described by Ngernmeesri and coworkers^[4d]^ in 2021.

Ngernmeesri and coworkers in 2021,^[4d]^ described the one‐pot nucleophilic aromatic substitution followed by Knoevanagel condensation and dehydration reactional steps to obtain the target compounds in a shorter reaction time, dibenzo[*b*,*f*]oxepines **4**, under a novel, simple, and transition‐metal‐free protocol (**Scheme** **44**). A total of 26 examples of desired dibenzo[*b*,*f*]oxepines **4** were synthesized in yields ranging from low to good (10%–72%) when 2‐methyl‐3‐nitrophenol **23** was reacted with a wide range of 2‐fluorobenzaldehydes **6** in the presence of 3.61 equiv. of Cs_2_CO_3_ as a base and DMF as the solvent at 120 °C in shorter reaction times (1–5 h). In general, it was not possible to relate the yields to the electronic and steric effects, only for some cases where relationship was noticeable, such as when aldehydes **6** substituted with EDG (fluoro, chloro, and bromo) at the ortho position were reacted under standard conditions. In these cases, the EWG accelerated the S_N_Ar reaction due to the enhanced reactivity the electrophilicity, of starting material (compounds **4z**, **4aa,** and **4ab**). A similar effect would be applied to starting materials **6** with EWG *para*‐substituted, however, it was not observed (compounds **4aj**, **4ak**, and **4al**). In addition, the compound was not obtained, probably due to competition in the S_N_Ar reaction caused by the presence of ortho and para fluoro‐substituted aldehyde as starting materials. Similar results were obtained for aldehydes substituted with strong EWG (NO_2_ and CF_3_ groups). In these cases, the desired compounds **4ap** and **4aq** were not obtained, and complex mixtures were formed in both cases. The target dibenzo[*b*,*f*]oxepines **4** containing strong EDG were obtained in poor yields for compounds **4ad** and **4ao** compared to **4ai** and **4au**, probably due to the presence of the OMe group in para or ortho to leaving group decreasing the electrophilicity of the aryl aldehyde in the S_N_Ar. In contrast, in the OMe group is attached to the meta position, this effect is minimized (compounds **4ai** and **4au**). The scope of phenols **23** was also checked under standard conditions, when 2‐methyl‐5‐substitutedphenols with NO_2_, −CN, or −CHO EWG groups were reacted with 2‐fluoro benzaldehyde **6c**. In these cases, the target products **4p**, **4av**, and **4aw** were synthesized in 69%, 28%, and 66% yield, respectively (Scheme 44). These results were probably due to the group's para about the methyl substituent, which increase the acidity of the benzylic hydrogen. This effect was not observed for the *meta*‐substituted precursors. In this case, the reaction did not work. Therefore, the protocol was successfully applied when the methyl group of starting material was changed to the ethyl group, in this case, the 11‐methyl‐3‐nitrodibenzo[*b*,*f*]oxepine **4ax** was isolated in 60% yield, a similar yield compared to the starting material without the methyl group (4p, 69%) (Scheme 44).

The total synthesis of the anticancer compound bauhinoxepin C **74a** was completed, yielding the target compound **74a** in 5.1% overall yield after seven steps starting from 2‐methyl resorcinol **23b**. In relation to this, after some changes, the method developed by the authors was applied in the synthesis of dibenzo[*b*,*f*]oxepines **4bc** (intermediate in the obtention of bauhinoxepin C **74a**). The 2‐methyl resorcinol **23b** was reacted with 2‐fluoro‐3,5‐dimethoxy‐4‐methylbenzaldehyde **6b** in the presence of 4.58 equiv. of K_2_CO_3_ in DMF as the solvent, at 160 °C for 8 h to obtain the desired compound **74a** in 59% yield (Scheme 44).

**Scheme 45:**
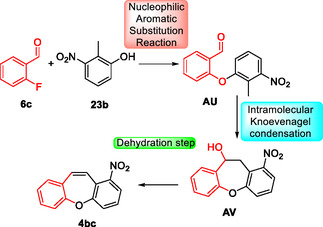
Reactional pathway proposed by Ngernmeesri and coworkers^[4d]^ in 2021.

The proposed reaction pathway starts with the formation of the biaryl ether **AU** from the reaction of 2‐methyl‐3‐nitrophenol **23b** with 2‐fluorobenzaldehyde **6c** in the presence of base to promote the intermolecular nucleophilic aromatic substitution reaction. Subsequently, the intramolecular Knoevenagel condensation occurs, leading to intermediate **AV**. The nitro group attached to the biaryl ether increases the acidity of the methyl group at the ortho position, favoring the reaction. Finally, the intermediate **AV** undergoes a dehydration step to obtain the designated dibenzo[*b*,*f*]oxepine **4bc** (**Scheme** **45**).

**Scheme 46:**
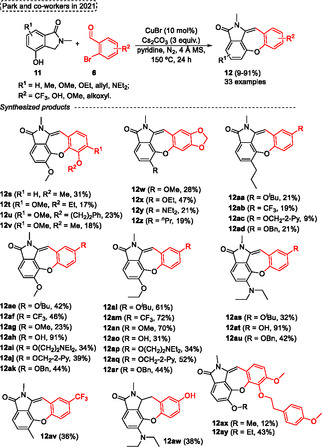
Synthesis of dibenzo[*b*,*f*]oxepine unit described by Park and coworkers^[^ ^46^ ^]^ in 2021.

Encouraged by the biological activities presented by benzoxepines compounds, in 2021, Park and coworkers^[^ ^46^ ^]^ reported a protocol to obtain hybrids of isoindolin‐1‐one and benzoxepines **12** from a one‐pot Cu‐catalyzed etherification/aldol condensation cascade reaction between several 4‐hydroxy‐2‐methylisoindolin‐1‐one **11** with different *o*‐bromobenzaldehydes **6** (**Scheme** **46**). The 4‐hydroxy‐2‐methylisoindolin‐1‐one **11** was prepared from different salicylaldehyde, which were reacted with benzyl bromide via nucleophilic substitution reactions to obtain the *O*‐benzyl protected compounds. Afterward, reductive amination was performed using methylamine and NaBH_4_, followed by ring cyclization in the presence of CO gas Cu/Pd to form the *O*‐benzyl‐2‐methylisoindolin‐2‐ones. Lastly, the 2‐methylisoindolin‐1‐one **11** was obtained after debenzylation of *O*‐Bn protective group using Pd/C in an H_2_ atmosphere. The target product **12** was synthesized by reacting of isoindolinones **11** with 2 equiv. of different 2‐bromobenzaldehydes **6** in the presence of 10 mol% of CuBr, 3 equiv. of Cs_2_CO_3_, using 4 Å molecular sieves (MS) in pyridine at 150 °C for 24 h. A total of 33 examples of the target dibenzoxepine lactam **12** were obtained in yields varying from low to excellent (9%–91%) under these conditions (Scheme 46). The biological activity of aristoyagonines synthesized was evaluated Showing a good inhibitory activity as well as a mode of action as bromodomain inhibitors, which can be used to treat cancer diseases. In this regard, the best results were obtained when substituents methoxy, ethoxy, ^*n*^propyl, or *N*,*N*‐diethylaminoethoxy group attached in the ring isoindolinones **11** were evaluated, did not have a significative difference in the inhibitory activities for these substituents. In contrast, better results were observed for derivatives of 2‐bromobenzaldehydes **6** when their structures contained hydroxyl or alkoxyl groups, which enhanced their inhibitory effects against Brd4 bromodomain (Scheme 46).

**Scheme 47:**
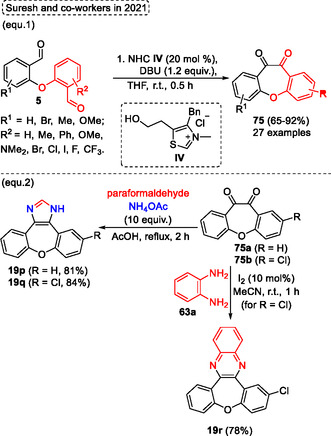
Synthesis of dibenzo[*b*,*f*]oxepine unit described by Suresh and coworkers^[^ ^47^ ^]^ in 2021.

Suresh and coworkers in 2021,^[^ ^47^ ^]^ applied the NHC‐catalyst for the synthesis of 1,2‐diketone functionalized dibenzo[*b*,*f*]oxepines **19** from an intramolecular benzoin condensation–oxidation reaction using 2,2′‐oxydibenzaldehyde **5** (**Scheme** **47**, eq. 1). The carbene‐catalyst (formed in situ) promotes the formation of a benzoin intermediate through the reaction of two aldehyde groups, which subsequently forms *α*‐*sec*‐hydroxyketone and after the oxidation step affords the target 1,2‐diketone‐oxepine core **75**. This protocol was efficient for a broad range of 2,2′‐oxydibenzaldehydes **5**, which were reacted in the presence of 20 mol% of NHC **IV**, 1.2 equiv. of DBU in THF as the solvent at r.t. for 0.5 h. Under these conditions, 27 examples of 1,2‐diketone containing dibenzo[*b*,*f*]oxepines **75** were synthesized in yields ranging from good to excellent (65%–92%) (Scheme 47, eq. 1). Subsequently, the synthesized 1,2‐diketones **75** were applied as starting material for further cyclization steps. When compounds **75a** and **75b** were reacted with paraformaldehyde in the presence of 10 equiv. of ammonium acetate in acetic acid at reflux temperature for 2 h the target imidazole‐fused dibenzoxepines **19p** and **19q** were obtained in good yields (81% and 84%, respectively). Additionally, the dibenzoxepine **75b** also reacted with *o*‐phenylenediamine **63a** in the presence of 10 mol% of I_2_. In this reaction MeCN was used as solvent at r.t., and after 1 h of reaction the target polycyclic compound, 7‐chlorodibenzo[2,3:6,7]oxepino[4,5‐*b*]quinoxaline **19r** was synthesized in a good yield (78%) (Scheme 47, eq. 2).

**Scheme 48:**
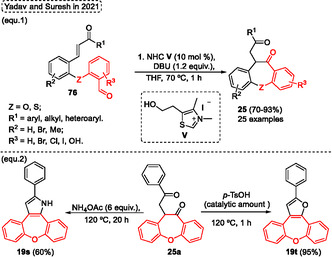
Synthesis of dibenzo[*b*,*f*]oxepine unit described by Yadav and Suresh^[^ ^48^ ^]^ in 2021.

However, in 2021, by altering the type of catalyst and reaction temperature, the same research group developed a highly robust intramolecular Stetter reaction of **76** using an NHC‐catalyst (**V**) to obtain the 1,4‐dicarbonyl substituted dibenzo[*b*,*f*]oxepine **25** (**Scheme** **48**, eq. 1).^[^ ^48^ ^]^ A broad range of substrate diversity was applied in the reaction using 10 mol% of NHC as a pre‐catalyst, 1.2 equiv. of DBU, THF as the solvent at 70 °C. After 1 h of reaction, a diverse range of dibenzo[*b*,*f*] oxepines **25** (25 examples) were obtained in yields ranging from good to excellent (70%–93%) (Scheme 48, eq. 1). The compound **25a** was applied to perform the Paal–Knorr reaction to obtain pyrrole‐ and furan‐dibenzo[*b*,*f*] oxepines fused compounds **19s** and **19t**. Firstly, the 1,4‐dicarbonyl compound **25a** was reacted in the presence of 6 equiv. of NH_4_OAc, at 120 °C for 20 h to synthesize the pyrrole‐dibenzo[*b*,*f*] oxepine **19s** with a yield of 60%. Similarly, when the same starting material **25a** was reacted in the presence of the catalytic amount of *p*‐TsOH at 120 °C for 1 h the furan‐dibenzo[*b*,*f*]oxepine **19t** was isolated in an excellent yield (95%) (Scheme 48, eq. 2).

**Scheme 49:**
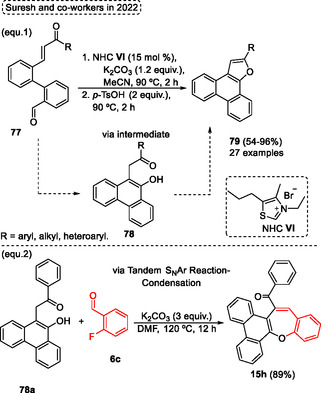
Synthesis of dibenzo[*b*,*f*]oxepine unit described by Suresh and coworkers^[^ ^49^ ^]^ in 2022.

In the next year (2022), the same research group used a carbene‐catalyzed intramolecular Stetter reaction followed by the Paal−Knorr reaction to obtain phenanthro[9,10‐*b*]furan **79**.^[^ ^49^ ^]^ The starting material **77,** containing the aldehyde and activated *α*,*β*‐unsaturated alkene unit attached in the ortho position, were reacted in a on‐pot two‐step protocol. The first step is performed in the presence of 15 mol% NHC **VI** catalyst and1.2 equiv. K_2_CO_3_ as the base, in MeCN as the solvent, at 90 °C for 2 h. Subsequently, in the intermediate key **78**, formed was added 2 equiv. of *p*‐TsOH and the mixture was reacted at 90 °C for 2 h (**Scheme** **49**, eq. 1). In contrast, the authors envisioned the formation of benzoxepine **15**, in this case, instead of formation of furan **79** from the intramolecular reaction of the key intermediate, this intermediate was reacted in the presence of 2‐fluorobenzaldehyde **6c** to perform a Tandem S_N_Ar reaction‐condensation. In this regard, the phenanthrene **78a** was reacted with 2‐fluorobenzaldehyde **6c** in the presence of 3 equiv. of K_2_CO_3_ as base in DMF as solvent at 120 °C for 12 h. Under these conditions, the target polycyclic aromatic compound **15h** containing oxygen/nitrogen atom, was synthesized in excellent yield (89%) (Scheme 49, eq. 2).

**Scheme 50:**
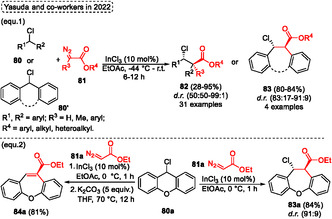
Synthesis of dibenzo[*b*,*f*]oxepine unit described by Yasuda and coworkers^[^ ^50^ ^]^ in 2022.

Also in 2022, Yasuda and coworkers^[^ ^50^ ^]^ reported the diastereoselective synthesis of *α*,*β*‐diaryl‐*β*‐haloesters **82** and **83** using a Lewis acid as the catalysts in the reaction of secondary halides with *α*‐diazo esters (**Scheme** **50**, eq. 1). This method involves the cleavage and the reformation of a carbon— chlorine bond, in this process the diastereoselectivity occurs in the key aryl‐migration step. In this regard, the protocol developed was efficient to several secondary halides **80** when reacted with different *α*‐diazo esters **81** in the presence of 10 mol% InCl_3_ in EtOAc, at temperatures ranging from −44 °C to r.t. for 6–12 h. Under these conditions, 31 examples of compounds **82** were obtained [28%–95% and *d.r.* (50:50–99:1] (Scheme 50, eq. 1). Furthermore, cyclic benzylic chlorides **80’** were used as starting materials. In this case, ring expansion to obtain fused polycyclic products **83** (4 examples) in better yields and diastereoselectivity [80%–84%, d.r. (83:17–91:9)] than those obtained from noncyclic benzylic chlorides. When the 9‐chloro‐9*H*‐xanthene was applied under standard conditions dihydrodibenzo‐[*b*,*f*]oxepine **80a** was obtained with 84% yield and excellent diastereoselectivity (91:9). This starting material **80a** was also used in a two‐step protocol. The first step was the same as described above, obtaining the **83a** in situ, followed by the addition of 5 equiv. of K_2_CO_3_, in THF at 70 °C. After 12 h of reaction the ethyl dibenzo[*b*,*f*]oxepine‐10‐carboxylate **84a** was obtained in good yield (81%) (Scheme 50, eq. 2).

**Scheme 51:**
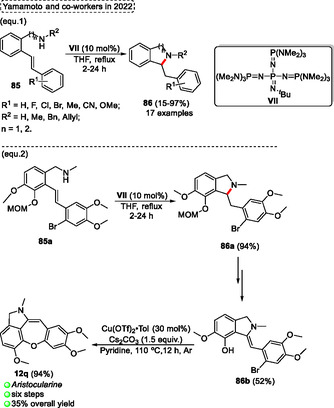
Synthesis of dibenzo[*b*,*f*]oxepine unit described by Yamamoto and coworkers^[^ ^51^ ^]^ in 2022.

Yamamoto and coworkers,^[^ ^51^ ^]^ in 2022, described the synthesis of isoindoline **86** from the reaction of amino alkenes **85** by intramolecular hydroamination using phosphazene **VII** as a catalyst. This method involved the reaction of several amino alkenes **85** in the presence of 10 mol% of catalyst **VII**, using THF as the solvent at reflux temperature, for reaction times ranging from 2 h to 24 h. Under these conditions, 17 examples of *N*‐heterocycles compounds **86** were obtained, with a broad functional group tolerance (such as halide, cyano, and methoxy groups) in yields ranging from low to excellent (15%–97%) (**Scheme** **51**, eq. 1). When the compound **85a** was applied under standard conditions, the target *N*‐heterocycle compound **86a** was obtained in excellent yield (94%) (Scheme 51, eq. 2). This compound was used as starting material **86a** to obtain the *Aristocularine* **12q**, which was synthesized in six steps with 35% overall yield. The target natural compound **12q**, containing an oxepine core, was obtained in excellent yield (94%) after the Ullmann coupling reaction, in which the compound **86b** was reacted in the presence of 30 mol% copper catalyst, 1.5 equiv. of Cs_2_CO_3_ as the base in pyridine as solvent at 110 °C for 12 h under Ar atmosphere (Scheme 51, eq. 2).

**Scheme 52:**
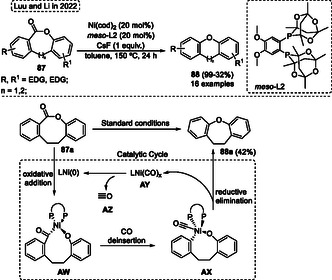
Synthesis dihydrodibenzooxepine **88** and mechanism proposed by Luu and Li^[^ ^52^ ^]^ in 2022.

In 2022, Luu and Li^[^ ^52^ ^]^ reported the formation of cyclic diaryl ethers **88** by a nickel‐catalyzed decarbonylation of lactones **87** (**Scheme** **52**). In this protocol, nickel(II) complexes were used to perform the challenging C(_sp2_)–O(aryl) reductive elimination. Several lactones **87** were reacted in the presence of Ni(cod)_2_ (20 mol%) as the catalyst and meso‐L2 (20 mol%) as the ligand, using 1 equiv. CsF in toluene as solvent at 150 °C for 24 h. Under these conditions, a wide range of cyclic diaryl ethers **88** (16 examples) were synthesized in yields ranging from low to excellent (32%–99%) (Scheme 52).^[^ ^52^ ^]^

When the starting material **87a** was reacted under standard conditions, the target dihydrodibenzooxepine **88a** was formed in moderate yield (42%) (Scheme 52). The mechanism proposed by the authors to access this compound **88a** starts with the oxidative addition of Ni‐complex into the C—O bond, forming the intermediate **AW**. Subsequently, after the CO deinsertion step, the intermediate **AX** is formed, which, after the reductive elimination step, affords the desired dihydrodibenzooxepine **88a** and the inactive complex L‐Ni(CO)x **AY**. This complex suffers a CO elimination **AZ** to regenerate the complex‐niquel catalyst to a new cycle (Scheme 52).

### 2.4 Protocols for Synthesizing Dibenzo[*b*,*f*]oxepine from 2023 to 2024

**Scheme 53:**
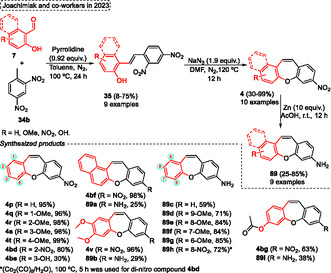
Synthesis of dibenzo[*b*,*f*]oxepine unit described by Joachimiak and coworkers^[^ ^53^ ^]^ in 2023.

Based on the results of the cytotoxic activity of dibenzo[*b*,*f*]oxepines previously synthesized **4** or **5**, advances in the synthesis, molecular docking, antiproliferative activity, and immunofluorescence analyzes of new stilbenes and dibenzo[*b*,*f*]oxepines **4** or **5** were described by Joachimiak and coworkers in 2023.^[^ ^53^ ^]^ The newdibenzo[*b*,*f*]oxepines **4** or **5** containing hydroxy, methoxy, nitro, and amine groups were synthesized and tested for cytotoxicity in cancer cell lines (HCT116 and MCF‐7) (**Scheme** **53**). A three‐step synthetic route was designed to obtain the targeted dibenzo[*b*,*f*]oxepines **4** or **5**, which starts from different 2‐hydroxyaldehydes **7** and 2,4‐dinitrotoluene **34b** reacting with 0.92 equiv. of pyrrolidine as a catalyst, using toluene as a solvent under a N_2_ atmosphere at 100 °C for 24 h. Under these conditions, 9 examples of stilbenes **35** were obtained in yields ranging from 8% to 75%. Compounds **35** were the starting materials to obtain the 3‐nitrodibenzo[*b*,*f*]oxepines **4** by performing a reaction in the presence of 1.9 equiv. of NaN_3_, DMF as the solvent, N_2_ atmosphere. After 12 h of reaction at 120 °C, ten examples of 3‐nitrodibenzo[*b*,*f*]oxepines **4** were synthesized, generally, in excellent yields (80%–99%). However, for starting materials containing hydroxy or acetyl groups, the yields were poor, with products **4be** and **4bg** affording yields of 30% and 63%, respectively. Focusing on obtaining amino compounds, the 3‐nitrodibenzo[*b*,*f*]oxepines **4** had the nitro group reduced through reaction using 10 equiv. of Zn, in AcOH at r.t. for 12 h. Under these conditions, nine examples of 3‐aminedibenzo[*b*,*f*]oxepines **89** derivatives were obtained in most cases significantly lower yields (25%–85%) compared to the nitro target compounds **4**. Interestingly, the dibenzo[*b*,*f*]oxepines **4bd** containing two nitro groups, could be selectively applied monoreduction (only one nitro group) using dicobalt octocarbonyl in water (Scheme 53).

**Scheme 54:**
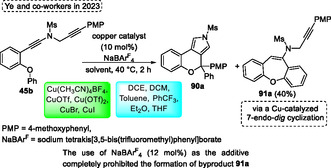
Synthesis of dibenzo[*b*,*f*]oxepine unit described by Ye and coworkers^[^ ^54^ ^]^ in 2023.

In 2023, Ye and coworkers^[^ ^54^ ^]^ described the first [1,2]‐Stevens‐type rearrangement via C_(sp2)_—O bond cleavage. In addition, vinyl cations promoting the first [1,2]‐aryl migration reactions, leading to the synthesis of chromeno[3,4‐*c*]pyrroles **90** (**Scheme** **54**). This protocol obtains the desired product **90** with high yields and enantioselectivity. However, during the optimization process when the starting material **45b** was reacted with different Cu‐catalysts and solvents, the compound **91a** was obtained as a side‐product. The oxepin **91a** was obtained via the 7‐endo‐*dig* cyclization reaction, to avoid this problem NaBAr^F^_4_ was used as an additive in the reaction protocol. After some studies, the authors found that the use of this additive completely inhibited the formation of the by‐product **91a** (Scheme 54).

**Scheme 55:**
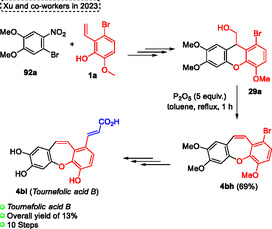
Synthesis of dibenzo[*b*,*f*]oxepine unit described by Xu and coworkers^[^ ^55^ ^]^ in 2023.

Given the importance of *Tournefolic acid B* (TAB) compound, since it is a potent component against myocardial ischemia reperfusion injury, Xu and coworkers^[^ ^55^ ^]^ reported in 2023 the synthesis, structure‐activity relationship as well as the cardioprotective activity of these compounds containing an oxepines core (**Scheme** **55**). In this regard, the compounds 1‐bromo‐4,5‐dimethoxy‐2‐nitrobenzene **92a** and 3‐bromo‐6‐methoxy‐2‐vinylphenol **1a** were used as starting materials for the total synthesis of TAB **4bi**, in which the target product **4bi** was obtained in an overall yield of 13% after ten steps. The key step for obtaining the oxepine unit occurred through ring expansion method of (1‐bromo‐4,6,7‐trimethoxy‐9* H*‐xanthen‐9‐yl)methanol **29a** compound, catalyzed by a Lewis acid. This compound was subjected to a reaction in the presence of phosphorus oxide (5 equiv.) in toluene as a solvent at reflux temperature for 1 h. Under these conditions, the expanded ring key oxepine **4bh** was obtained with a 69% yield (Scheme 55).

**Scheme 56:**
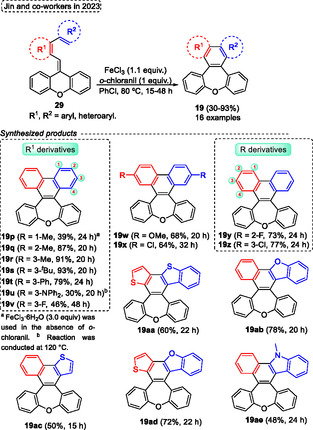
Synthesis of dibenzo[*b*,*f*]oxepine unit described by Jin and coworkers^[^ ^56^ ^]^ in 2023.

In 2023, Jin and coworkers^[^ ^56^ ^]^ reported the tandem oxidative ring expansion (TORE), which promotes the six‐ to seven‐membered ring of 9‐(biaryl‐2‐ylmethylene)‐9*H*‐xanthenes **29** to dibenzo[*b*,*f*]phenanthro[9,10‐*d*]oxepines **19** (**Scheme** **56**). This strategy combines *o*‐chloranil and FeCl_3_ to mediate TORE reaction. The *o*‐chloranil undergoes the unexpected [4 + 2] cycloadduct with xanthenes **29**, which subsequently undergoes suffers an intramolecular Friedel−Crafts cyclization, forming relatively stable cation and radical species to give expanded ring efficiently (Scheme 56).

The TORE protocol involves the reaction of several 9‐(biaryl‐2‐ylmethylene)‐9H‐xanthenes **29** in the presence of 1.1 equiv. of FeCl_3_ and 1 equiv. of *o*‐chloranil in PhCl as the solvent at 80 °C. Under these conditions, 16 examples of the target compounds **19** were obtained, with yields varying from low to excellent (30%–93%) after reaction times of 15–48 h. This approach was efficiently applied to EWG and EDG groups attached to the R^1^ aromatic ring and to neutral (H) and EWG (F, Cl) bonded in the romantic ring R. The symmetrical dibenzo[*b*,*f*]phenanthro[9,10‐*d*]oxepines **19w** and **19x**, containing methoxy group and chlorine atoms, were synthesized with similar yields of 68% and 64%, respectively. Additionally, starting materials containing *O*‐, *S*‐, *N*‐heterocycles in the R or R^1^ were subjected to standard conditions, yielding the desired dibenzo[*b*,*f*]phenanthro[9,10‐*d*]oxepines **19aa**, **19ab**, **19ac**, **19ad**, and **19ae** with yields of 60%, 78%, 50%, 72%, and 48%, respectively (Scheme 56).

**Scheme 57:**
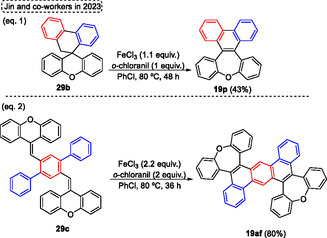
Synthesis of poli‐dibenzo[*b*,*f*]oxepine unit described by Jin and coworkers^[^ ^56^ ^]^ in 2023.

The protocol was further extended to obtain polyaromatic substituted oxepines **19**, in these cases, the starting materials were subjected to optimized reaction conditions, affording the compounds **19p** and **19af** in yields of 43% and 80%, respectively. Notably, eq. (2) of **Scheme** **57** uses 2.2 equiv. of FeCl_3_ and 2 equiv. of *o*‐chloranil due to the symmetry of starting material **29c**, contains two reactive units to undergo the TORE process (Scheme 57).

**Scheme 58:**
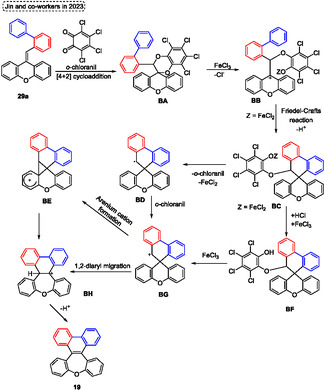
Mechanism proposed by Jin and coworkers^[^ ^56^ ^]^ in 2023.

The proposed mechanism starts with the hetero [4 + 2] cycloaddition reaction between starting material **29a** with *o*‐chloranil to afford the cyclic intermediate **BA**, which, in the presence of a Lewis acidic oxidant FeCl_3_, ring opening occurs, yielding the cation intermediate **BB**. Subsequently, the key spirocycle **BC** formes by the intramolecular Friedel−Crafts cyclization reaction of this intermediate **BB**, in the next step, the mechanism splits into two reactional pathways, the firstly occurs by the formation of spirocyclic radical **BD** (by the homolytic cleavage of the C—O bond) followed by oxidation using *o*‐chloranil give the cation intermediate **BG**. The same intermediate **BG** can also be generated through an alternative pathway, which occurs the protonation of **BC** followed by action Lewis acidic (FeCl_3_) in the remotion of the tethered tetrachlorophenol unit. An intramolecular rearrangement occurs in the intermediate **BE** to afford the intermediate **BH** by the formation of arenium cation, as well as by the direct 1,2‐aryl migration of **BG**. Lastly, the target product **19** forms after the final deprotonation step of the intermediate **BH** previously (**Scheme** **58**).

**Scheme 59:**
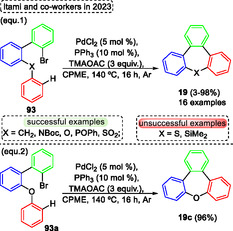
Synthesis of dibenzo[*b*,*f*]oxepine unit described by) Itami and coworkers^[^ ^57^ ^]^ in 2023.

Recently (2023), Itami and coworkers^[^ ^57^ ^]^ described a Pd‐ catalyzed protocol to synthesize seven‐membered rings of polycyclic aromatic compounds **19** through coupling intramolecular reaction by C–H activation. This efficient protocol tolerated a wide range of (hetero)arene‐fused bromo(hetero)arenes **93** containing methylene, nitrogen, phosphorus, oxygen, and sulphone as heteroarenes units (X = CH_2_, NBoc, O, POPh, SO_2_). A total of 16 examples (hetero)arenefused heptagonal compounds **19** were synthesized in yields ranging from low to excellent (3%–98%) when the starting materials **93** were reacted in the presence of 5 mol% of PdCl_2_ as catalyst, 10 mol% of PPh_3_ as ligand, and 3 equiv. of tetramethylammonium acetate (TMAOAC) in cyclopentyl methyl ether (CPME) at 140 °C for 16 h, under Ar atmosphere (**Scheme** **59**, eq. 1). Gratifyingly, the 2‐bromo‐2′‐phenoxy‐1,1′‐biphenyl **93a** was efficiently applied under standard conditions, affording the desired tribenzo[*b*,*d*,*f*]oxepine **19c** in excellent yield (96%). In contrast, the protocol proved to be sensitive to S and SiMe2‐tethered substrates, in these cases the reaction did not proceed (Scheme 59, eq. 2).

**Scheme 60:**
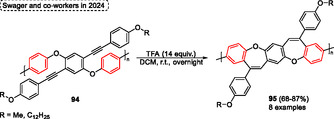
Synthesis of dibenzo[*b*,*f*]oxepine unit described by Swager and coworkers^[^ ^58^ ^]^ in 2025.

Currently, Swager and coworkers^[^ ^58^ ^]^ designed and synthesized *π*‐conjugated ladder/step‐ladder polymers (**Scheme** **60**). This uninterrupted ring‐fused polymer developed has great interest due to the high extended *π*‐conjugation, as well as due to the unique proprieties, that make them with great potential applications as OLEDs. In this protocol, several alkyne‐containing polys (arylene ether) **94** were used as starting materials in the intramolecular aromatic electrophilic cyclization reaction promoted by trifluoroacetic acid (TFA) in DCM at r.t. Under these conditions, several oxepine‐based polymers **95** (8 examples) were synthesized and also characterized. Typically, polymers are composed of five‐ and six‐membered rings; however, this novel class of polymers **95** incorporates 7‐membered rings, which exhibit a large Stokes shift between the lowest energy absorption maximum and the emission maximum. This effect likely arises from the photoinduced planarization that occurs in the excited state, in which these compounds display enhanced aromatic stabilization energy according to Baird's rule” (Scheme 60). Over the years, other work has also reported the synthesis of more complex molecules containing the **95** unit, as well as the photophysical study of these compounds.^[^ ^59^ ^]^

**Scheme 61:**
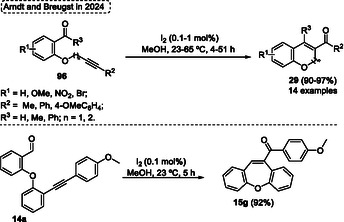
Synthesis of dibenzo[*b*,*f*]oxepine unit described by Arndt and Breugst^[^ ^60^ ^]^ in 2024.

Arndt and Breugst (2024)^[^ ^60^ ^]^ reported the intramolecular reaction of starting materials containing aldehyde or ketone and alkyne functional groups via a carbonyl‐alkyne RCM reaction. Using this strategy, the *α*,*β*‐unsaturated cyclic carbonyls compounds can be synthesized with small amounts of molecular iodine (0.1–1 mol%). as catalyst (**Scheme** **61**). The best conditions identified by the authors involve reacting the starting materials **96**, in the presence of I_2_ (0.1–1 mol%) and MeOH as a solvent, at temperatures and time of reaction ranging from 23 °C and 4 h to 65 °C and 51 h. Under these conditions, 14 examples of target compound **29** were obtained in excellent yields (90%–97%), this protocol was not sensitive to electronic effect and tolerated a wide range of functional groups, such as CN, CO_2_Me, Br and OH). Interestingly, the reaction proceeded efficiently only in the presence of MeOH as a solvent, due to the high polarity of this compound, this can act in the solvation of all charged intermediates, as well as in the formation of the iodonium key intermediates (I^+^ or [MeOHI]^+^) as a Lewis basic solvent (Scheme 61). Subsequently, when these previously established conditions were applied to starting material **14a**, using 1 mol% of I_2_ as a catalyst, MeOH as a solvent, the desired dibenzo[*b*,*f*]oxepin‐10‐yl(4‐methoxyphenyl)methanone **15g** was obtained in 92% of yield, after 5 h of reaction at 23 °C (Scheme 61).

**Scheme 62:**
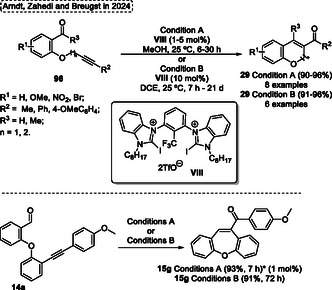
Synthesis of dibenzo[*b*,*f*]oxepine unit described by Arndt, Zahedi, and Breugst^[^ ^61^ ^]^ in 2024.

Similarly to the previously described protocol, the same research group (Arndt, Zahedi, and Breugst) reported, in 2024,^[^ ^61^ ^]^ reported the halogen‐bond catalyzed protocol in the carbonylalkyne metathesis reaction to form the cyclic or not *α*,*β*‐unsaturated carbonyls desired compounds **29**. This protocol reports advances in the use of halogen‐bond catalysis instead of the use of molecular iodine catalysts as reported. Using this powerful strategy, the authors obtained two optimized reaction conditions, both using bidentate halogen bond donor catalysts. Conditions A uses 1–5 mol% of catalyst **VIII**, MeOH as solvent at 25 °C for times of reactions ranging from 6 to 30 h. Under these conditions, the target compounds **29** (eight examples) were synthesized, achieving excellent yields (90%–96%) (**Scheme** **62**).

Under condition B, MeOH was replaced with DCE as the solvent, as well as an increase to 10 mol% of catalyst **VIII** was required. In this case, this modification necessitated significantly longer reaction times, ranging from 7 h to 21 days, to give the same products **29** (6 examples) in similar yields (91%–96%). In this sense, when the starting material **14a** was reacted under the previously established conditions A or B, the target dibenzo[*b*,*f*]oxepin‐10‐yl(4‐methoxyphenyl)methanone **15g** was formed in excellent yields 93% (conditions A, 7 h, 1 mol% of **VIII**) and 91% (Conditions B, 72 h) (Scheme 62).

## 3 Conclusion

Over the past decade, remarkable advances have been witnessed in the synthesis of dibenzo[*b*,*f*]oxepines, driven by growing interest in their structural diversity and potential applications in medicinal chemistry and materials science. This review has outlined a wide range of synthetic methodologies—ranging from classical approaches to more innovative strategies—demonstrating the continuous evolution of this field. Key techniques, including cross‐coupling reactions, intramolecular cyclizations, and molecular diversification, have played a crucial role in expanding the synthetic toolbox available for the synthesis of these challenging scaffolds.

Beyond improvements in efficiency and selectivity, increasing attention to sustainability has shaped recent research, promoting the development of more eco‐friendly methods and the use of less toxic reagents. Nevertheless, challenges persist, including the need to broaden the structural diversity of dibenzo[*b*,*f*]oxepines accessible via synthesis and the optimization of reaction conditions to ensure high and reproducible yields.

Moving forward, interdisciplinary collaboration will be essential to address these challenges. The integration of computational modeling techniques and advanced structural analysis methods is crucial, and creative synthesis approaches hold promise for opening new avenues in the synthesis and application of dibenzo[*b*,*f*]oxepines. Through collective scientific efforts, the full potential of these versatile compounds can be realized, paving the way for impactful advancements across multiple scientific and technological domains.

## Conflict of Interest

The authors declare no conflict of interest.
